# Functional polymer materials containing heavy group‐14 elements focusing on germanium and tin

**DOI:** 10.1002/smo.20240064

**Published:** 2025-03-16

**Authors:** Masayuki Gon, Kazuo Tanaka

**Affiliations:** ^1^ Department of Polymer Chemistry Graduate School of Engineering Kyoto University Kyoto Japan; ^2^ Department of Technology and Ecology Graduate School of Global Environmental Studies Kyoto University Kyoto Japan

**Keywords:** germanium, heavy element, polymer, tin

## Abstract

In this review, the synthesis, functions, and applications of the polymers containing germanium and tin, which are heavy group 14 elements, in their polymer frameworks are summarized. Germanium and tin can form similar skeletal structures with their homologues carbon and silicon, whereas the polymers containing germanium and tin show unique properties derived from their large atomic radii and weak binding energies. For example, polygermane and polystannane exhibited light absorption in the UV–visible region and conductivity because of the σ‐conjugation through the polymer main‐chain constructed by σ‐bonds between heavy elements. The σ‐conjugation was affected by the conformational change of the polymer main‐chain, and thermochromic properties can be induced. Furthermore, the weak bonds were able to be cleaved homolytically upon photoirradiation, and radicals were subsequently generated. By incorporating hypervalent heavy elements into the π‐conjugated system, it was possible to modulate the electronic structures of the π‐conjugated system through σ*–π* conjugation with highly coordinated elements. Finally, applications for organic solar cells, organic light‐emitting materials, and chemical sensors have been achieved. Herein, representative synthetic methods and unique properties for creating smart materials with germanium and tin will be explained.

## Graphical abstract

The synthesis, functions, and applications of the polymers containing heavy group 14 elements focusing on germanium and tin are outlined. Owing to their large atomic radii and weak binding energies, the polymers show unique properties: chromism, luminescence, carrier mobility, and reactivity to produce radials and higher coordinated states. These properties are also characterized by the σ‐conjugation, σ*–π* conjugation, and hypervalent states, which are applied for sensors, photoinitiators, lithographic techniques, organic solar cells, and organic light‐emitting materials. The polymers incorporating heavy elements should be one of the promising candidates for smart materials.

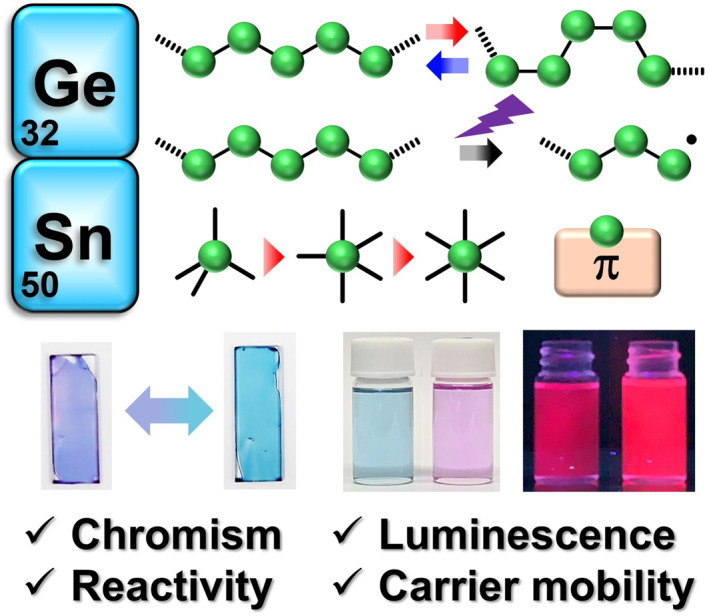

## 1 INTRODUCTION

Most polymers are composed of a carbon (C) skeleton and are highly designed to perform desired functions depending on their structures through various chemical reactions involving bond recombination of four stable chemical bonds.[^1^, ^2^] Thus, by molding and processing various materials, a wide variety of products have been made, ranging from conventional plastics to innovative optoelectronic devices. Organosilicon (Si) polymers, which consist of the same group 14 elements as carbon, have also been applied to high‐performance polymers owing to their high durability and flexibility.[^3^, ^4^] Historically, much research has been done on what functions can be achieved by converting elements heavier than silicon, such as germanium (Ge), tin (Sn), and lead (Pb). With the development of synthetic and analytical technologies, the exploration of functional materials using the inherent properties of the elements continues to evolve, and studies on elements are attracting much attention in various fields. From these backgrounds, this review will focus on the synthesis and characteristics of polymers containing these heavy elements, especially germanium and tin.

First, the fundamental nature of group 14 elements is discussed. The single‐bond covalent radii of group 14 elements are reported as 0.773 Å for C, 1.176 Å for Si, 1.225 Å for Ge, 1.400 Å for Sn, and 1.441 Å for Pb.^[5]^ Bond dissociation energies (BDEs) in diatomic molecules are evaluated to be 618.3 kJ/mol for C−C, 310 kJ/mol for Si−Si, 264.4 kJ/mol for Ge−Ge, 187.7 kJ/mol for Sn−Sn, and 86.6 kJ/mol for Pb−Pb.^[6]^ BDEs for single σ‐bonds are estimated to be 346 kJ/mol for C−C, 222 kJ/mol for Si−Si, 188 kJ/mol for Ge−Ge, 146 kJ/mol for Sn−Sn.^[7]^ These data suggest that germanium and tin have weak covalent bonds characterized by long bond lengths, and that they are more sensitive to stimuli such as light and nucleophilic attack. In the case of lead, a very limited number of the compounds including Pb–Pb bonds have been reported due to the smallest bond energy.^[8]^ On the other hand, it has been reported that the BDE of Me_3_Sn−SnMe_3_ is 257.7 kJ/mol and that of Et_3_Sn–SnEt_3_ is 354 kJ/mol, which is close to that of Me_3_C−CMe_3_ (377.4 kJ/mol).[^7^, ^9^] Thus, substituents can reinforce the strength of weak covalent bonds involving heavy elements.

The σ orbitals to construct σ‐bonds in polysilanes, polygermanes, and polystannanes are delocalized throughout the framework, referred to as σ‐conjugation.[^10^, ^11^] It is theoretically suggested that the degree of electron delocalization through σ‐conjugation should be affected by the conformation of the polymer main‐chain.^[12]^ In addition, it is known that the polymers exhibit light absorption in the UV–visible region and have conductivity as semiconductors.^[13]^ Furthermore, substitution of carbon with silicon, germanium, and tin can modulate the energy levels of carbon‐based ring‐fused π‐conjugated systems through σ*–π* conjugation,^[14]^ which can improve performance of organic photovoltaics (OPVs)^[15]^ and organic light‐emitting diodes (OLEDs).[^16^, ^17^]

Herein, we describe the synthetic methods and characteristics of germanium‐ and tin‐containing polymers. The uniqueness and potentiality of these polymers for functional materials are discussed as well as how they can lead to the development of next‐generation materials. Recently, we also summarized the effects of heavy p‐block elements on the photophysical properties of π‐conjugated complexes and organoelement compounds.^[18]^ Therefore, for more information on the influence of elements in small compounds, please refer to that literature.

## 2 EXAMPLES OF HEAVY GROUP‐CONTAINING POLYMERS

### 2.1 Germanium (Ge)‐containing polymers

#### 2.1.1 Polygermanes

**SCHEME 1:**
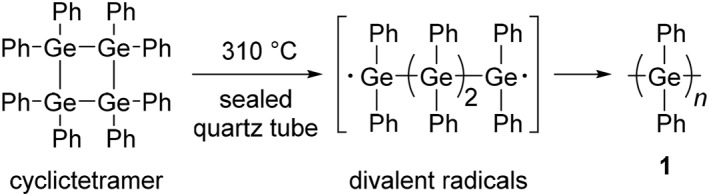
Synthesis of a polygermane by ring‐opening polymerization (ROP).

**SCHEME 2:**
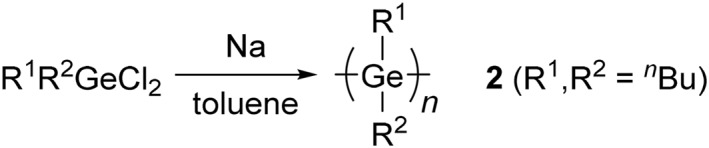
Synthesis of a polygermane by a Wurtz‐type coupling reaction.

**SCHEME 3:**
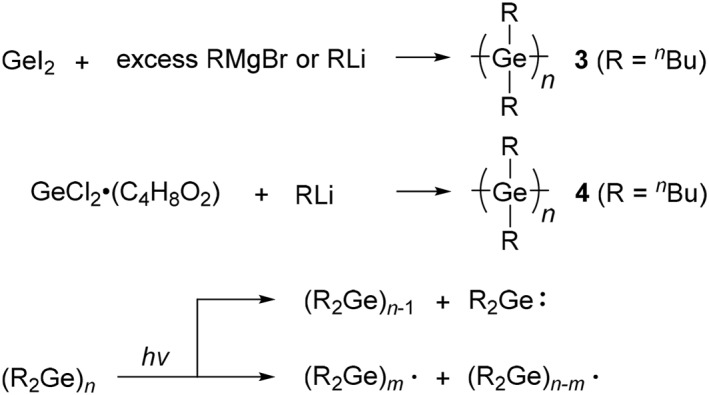
Synthesis of polygermanes with germylenes, and photolysis of polygermanes.

**SCHEME 4:**
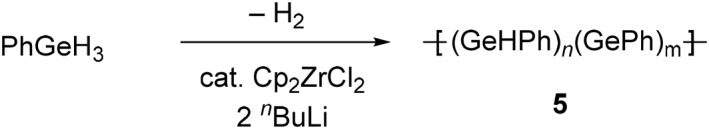
Synthesis of polygermanes by a metal‐catalyzed dehydrogenative coupling reaction.

**SCHEME 5:**
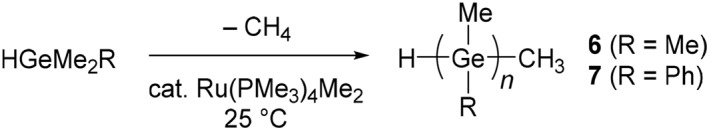
Synthesis of polygermanes by a metal‐catalyzed demethanative coupling reaction.

**SCHEME 6:**
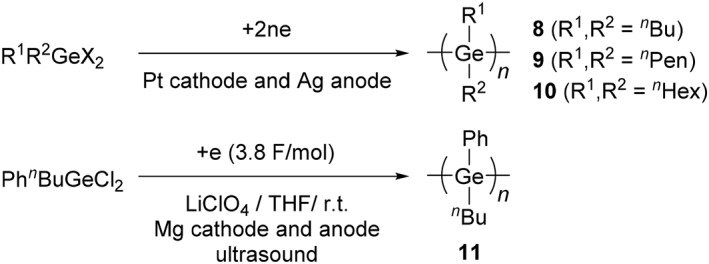
Synthesis of polygermanes by an electroreductive synthesis.

**SCHEME 7:**
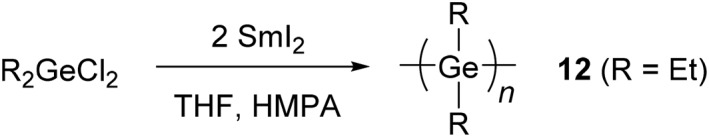
Synthesis of polygermanes by an electroreductive synthesis.

**SCHEME 8:**
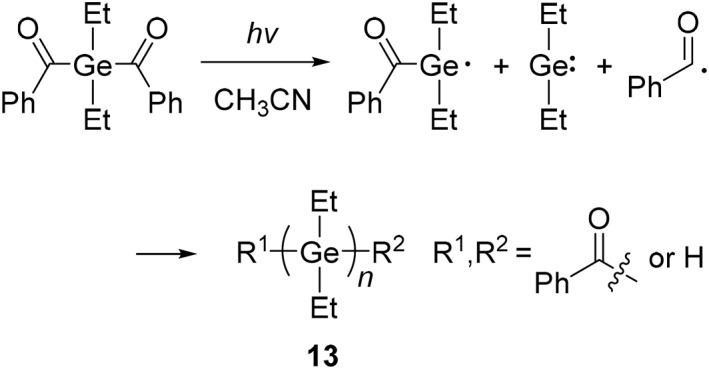
Synthesis of polygermanes by a photochemical synthesis.

**SCHEME 9:**
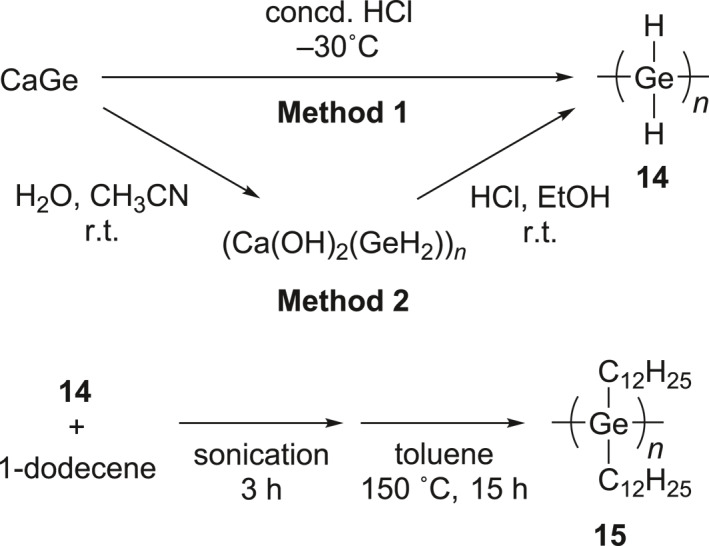
Synthesis of polygermanes by a topotactic deintercalation.

**FIGURE 1:**
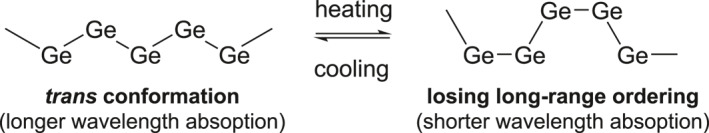
Conformational change of polygermanes depending on the temperature.

Polygermanes can be regarded as a germanium analog of polyolefins and have a polymer backbone catenated with Ge atoms. The first discovery of organogermanium compounds with Ge−Ge bonds was in 1925, and research on polygermanes has increased since the 1980s mainly owing to the development of synthetic methods.[^19^, ^20^] Polygermanes have been synthesized by various types of reactions such as a ring‐opening polymerization (ROP),[^21^, ^22^] a Wurtz‐type coupling reaction,[^23^, ^24^, ^25^, ^26^, ^27^, ^28^] a reaction of germylenes,[^26^, ^29^] a metal‐catalyzed dehydrogenative[^30^, ^31^] or demethanative[^32^, ^33^, ^34^] coupling reaction, an electroreductive reaction,[^35^, ^36^, ^37^, ^38^, ^39^, ^40^] a one‐electron reduction reaction,[^41^, ^42^] a photochemical reaction,^[43]^ and a topotactic deintercalation reaction^[44]^ (Schemes 1, 2, 3, 4, 5, 6, 7, 8, 9, Figure 1, and Table 1). Polygermanes exhibit light absorption bands around 300–380 nm originating from σ‐conjugation between the continuous Ge−Ge σ‐bonds. In addition, since the absorption property depends on the conformation of the main‐chain polygermanes, they show discontinuous thermochromism.[^24^, ^26^, ^39^, ^47^, ^48^] Below the transition temperature, the backbone prefers all‐*trans* conformation guided by side‐chain interaction and the absorption bands are observed in the longer wavelength region (ca. 370 nm). Above the transition temperature, on the other hand, the long‐range order of the polymer main‐chain is lost by thermal motion, and the absorption bands are detected in the shorter wavelength region (ca. 330 nm).[^48^, ^49^] The contribution of σ‐conjugation to the light absorption energy has been revealed by theoretical calculations.[^50^, ^51^, ^52^] In general, the absorption bands of polygermanes (Ge) are located in the longer wavelength region than those of polysilanes (Si) and in the shorter wavelength region than those of polystannanes (Sn).^[13]^ Moreover, homolytic cleavage of Ge−Ge bonds proceeds upon photoirradiation followed by the generation of germanium radicals.[^26^, ^53^, ^54^, ^55^, ^56^] These photodegradation behaviors can be applicable to lithography.[^57^, ^58^] High molecular‐weight polygermanes have lower ionic potentials than polysilanes,[^59^, ^60^] and the polygermanes effectively induce electron transfer to acceptors.[^61^, ^62^] The copolymers of germanes with silanes or stannanes can also be prepared, and their optical and physical properties vary depending on the components.[^27^, ^35^, ^63^, ^64^, ^65^, ^66^] These functions can be also modulated by the selection of side‐chains, and a various types of polygermanes have been synthesized.

**TABLE 1:** Data and properties of polygermanes (GeR_2_)_*n*_.

	R	Synthetic method	*M*_n_	*M*_w_	Properties	References
**1**	Ph	Ring‐opening polymerization	11,300	−	−	[22]
**2**	^*n*^Bu	Wurtz‐type coupling	423,000	736,000	Light absorption (*λ*_abs_ = 333 nm) Photoreactivity	[23]
**3**	^*n*^Bu	Germylene	1800	2200	Light absorption Thermochromism Photoreactivity	[26]
**4**	^*n*^Bu	Germylene	6200	17,900	Light absorption (*λ*_abs_ = 324 nm) Photoreactivity	[29]
**5**	H or Ph	Metal‐catalyzed dehydrogenative coupling	20,000	44,000	Light absorption (*λ*_abs_ = 329 nm) Partial network structures	[31]
**6**	Me	Metal‐catalyzed demethanative coupling	5000	144,000	−	[32]
**7**	Me and Ph	Metal‐catalyzed demethanative coupling	7900	6500	Light absorption (*λ*_abs_ = 332 nm)	[33]
**8**	^*n*^Bu	Electroreductive synthesis	2000	14,000	Light absorption	[37]
**9**	^*n*^Pen	Electroreductive synthesis	3900	11,000	(*λ*_abs_ = 325 nm for **8**)	
**10**	^*n*^Hex	Electroreductive synthesis	6100	10,000	(*λ*_abs_ = 327 nm for **9**) (*λ*_abs_ = 325 nm for **10**) Thermochromism	
**11**	^*n*^Bu and Ph	Electroreductive synthesis	19,900	−	−	[39]
**12**	Et	One‐electron reduction	4330	4890	Light absorption (*λ*_abs_ = 325 nm)	[42]
**13**	Et	Photochemical synthesis	2750	3050	Light absorption Photoreactivity Macrophotoinitiator	[43]
**14**	H	Topotactic deintercalation of CaGe	37,000	−	Precursor of substituted polygermanes	[44]
**15**	Dodecyl	Thermally induced hydrogermylation	500,000–700,000	−	−	
**16**	Si(OMe)_3_ and Me	Metal‐catalyzed demethanative coupling	13,900	18,100	Light absorption and emission (*λ*_abs_ = 332 nm, *λ*_PL_ = 369 nm)	[34]
**17**	Teteraphenyl‐germole	Wurtz‐type coupling	4400	4600	Light absorption and emission (*λ*_abs_ = 368 nm, *λ*_PL_ = 499 nm) Detection of explosives	[45]
**18**	Dithieno‐germole	Wurtz‐type coupling	4600	11,000	Light absorption and emission (*λ*_abs_ = 260, 380 nm, *λ*_PL_ = 431 nm, *Φ*_PL_ = 5%)	[28]
**19**	Cyclopentene	Metal‐catalyzed dehydrogenative coupling	−	15,000−24,000	Photopatterning of Ge metal for semiconductors (*λ*_abs_ = 292 nm)	[46]

In the early days of the research of polygermanes, it was discovered that poly(diphenylgermane) (**1**) was produced by ROP of octaphenylcyclotetragermane (Scheme 1).^[22]^ The formation mechanism of polygermane is based on three steps: 1. formation of divalent radicals by heating, 2. transport of these radicals, and 3. addition polymerization on a cold surface. First, the amorphous polymer was prepared by heating (310°C, 10 h) octaphenylcyclotetragermane and cooling in a vacuumed quartz tube. After the additional heating (285°C, 10 h) and cooling in the colder zone (250–260°C), polymer **1** was obtained as a prismatic crystal. From the vapor phase osmometry, the number‐average molecular weight (*M*_n_) of the crystalline polymer **1** was determined to be 11,300. The generation of polygermanes with ROP is interesting; however, this method has hardly been used as the common synthetic strategy for polygermanes. This is because the severe reaction condition might cause decomposition of thermally reactive side‐chains such as alkyl groups. As another synthetic method of polygermanes, a glow discharge polymerization technique was conducted.^[67]^ The colorless and transparent polymer films were able to be prepared from tetramethylgermanium monomer; however, the polymer included the reacted germanium components composed of Ge−C, Ge−O−C, and Ge−O−Ge bonds in addition to germanium metal.

Poly(di‐*n*‐butylgermanes) (**2**), the first high molecular weight, soluble, formable and easy to handle polygermanes, was reported to be synthesized by a Wurtz‐type coupling reaction of di‐*n*‐butyldichlorogermanes in toluene (Scheme 2).^[23]^ Bimodal peaks of polymer **2** were detected in the chart of gel permeation chromatography (GPC), and the high molecular‐weight fraction had *M*_n_ = 423,000 (*M*_w_/*M*_n_ = 1.74). Polymer **2** showed UV absorption, and the maximum absorption wavelength (*λ*_abs_) of it was 333 nm, which was longer than that of the silicon analog, poly(di‐*n*‐butylsilane) (*λ*_abs_ = 314 nm), and shorter than that of the tin analog, poly(di‐*n*‐butyltin) (*λ*_abs_ ∼ 365 nm). It was also reported that the maximum absorption wavelengths can be tuned in the range from 300 to 350 nm by constructing copolymers of organosilicon and organogermanium monomers with various alkyl substituents.

Although the Wurtz‐type coupling reaction has been widely used in the preparation of polygermanes with a variety of side‐chains,[^24^, ^26^] there are several challenges to overcome with this reaction: it is dangerous due to the use of alkaline metals and the isolated yields of polymer products are relatively low (usually 10%–20%). Additionally, the polymers have broadened polymer dispersity indices (*M*_w_/*M*_n_) due to the bimodal GPC profile consisting of desired high molecular‐weight polygermanes and undesired low molecular‐weight cyclic byproducts. To solve these issues, the use of germylenes as a precursor of polygermanes was proposed.[^26^, ^29^] Two‐types of procedures are shown in Scheme 3. The first method is the use of a stable inorganic germylene precursor, diiodogermylene (GeI_2_), with a Grignard reagent or an organolithium reagent.^[26]^ In this reaction, the alkyl chains of organometallic reagents are introduced as the side‐chains of polygermanes. By using this method, poly(di‐*n*‐butylgermane) (**3**) with narrower molecular weight distribution (*M*_n_ = 1,800, *M*_w_/*M*_n_ = 1.22) was provided in higher yields (55%) than the Wurtz‐coupling methods, although the molecular weight was lower. The second method is based on ligand substitution polymerization of a 1,4‐dioxane complex of germanium dichloride with an organolithium reagent at low temperature.^[29]^ Divalent germanium halides (germylenes) can be prepared as stable species unlike divalent silicone halides (silylenes). The polymerization of the germylene and *n*‐butyllithium at −78°C provided poly(di‐*n*‐butylgermane) (**4**) (*M*_n_ = 6,200, *M*_w_/*M*_n_ = 2.88) in up to 98% yield; however, bimodal peaks were still detected in the GPC profiles. These methods are experimentally easier and safer than Wurtz‐type coupling reactions. In the Wurtz‐type coupling reaction, it was reported that the yields of polygermanes were improved up to 74% by reacting at low refluxing temperatures in diethyl ether in the presence of 15‐crown‐5.^[25]^

**FIGURE 2:**
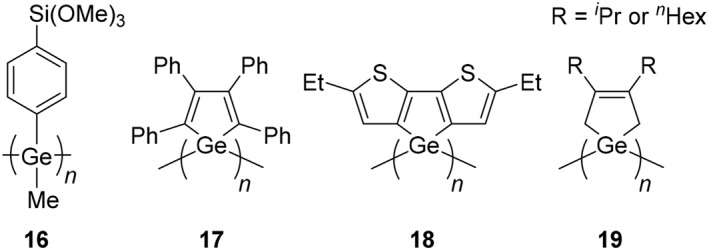
Structures of polygermanes for applications.

Interestingly, it is known that polygermanes shows thermochromism.^[26]^ For example, poly(di‐*n*‐hexylgermane) (^*n*^Hex_2_Ge)_*n*_ has an absorption band at 340 nm at room temperature, whereas the band is bathochromic shifted to 350 nm at −60°C. Poly(di‐*n*‐butylgermane) (^*n*^Bu_2_Ge)_*n*_ also exhibits thermochromism. This thermochromism, as with polysilanes, reflects a conformational change in which the proportion of *trans* conformations increases as temperature decreases (Figure 2). On the other hand, poly(diethylgermane) (Et_2_Ge)_*n*_ and poly(*n*‐hexylphenylgermane) (^*n*^HexPhGe)_*n*_ hardly show clear thermochromism. It is probable that the conformation of these polygermanes is fixed within the measured temperature ranges. Additionally, the thermochromic behavior of polygermane films have also been reported.[^23^, ^24^, ^48^] It is noted that thermochromic behavior of polygermanes with alkyl chains is observed at lower temperatures than that of polysilanes. The photolysis of polygermanes upon UV irradiation was also investigated.^[26]^ The main‐chain of polygermanes is homolytically cleaved upon UV irradiation accompanied by the generation of germanium radicals and germylenes (Scheme 3).

Dehydrogenative coupling polymerization of primary germanes is applicable for synthesizing polygermanes similarly to polysilanes.^[68]^ The polymerization of phenylgermane was performed in the presence of a catalytic amount of bis(cyclopentadienyl)zirconium(IV) dichloride (Cp_2_ZrCl_2_) and two equivalents of *n*‐butyllithium (Scheme 4).^[31]^ After reacting for 5 days, the viscosity of the mixture increased and trimodal peaks were detected in the GPC profile (*M*_n_ = 20,000 (*M*_w_/*M*_n_ = 2.2), 2600 (*M*_w_/*M*_n_ = 1.3), and 610 (*M*_w_/*M*_n_ = 1.1)). The mixture was purified by Florisil column and subsequent reprecipitation from THF solution to pentane to afford poly(phenylgermane) (**5**) with partially network structure in 67% isolated yield. The optical property was similar to the linear polygermane and thermogravimetric analysis (TGA) of the polymer under nitrogen hardly showed substantial weight loss up to 250°C. Similar polymerization for obtaining phenylgermane has been carried out by using a dimethyltitanocene catalyst, however main products were tetramers.^[30]^

A demethanative coupling reaction with ruthenium catalysts is known to be another efficient catalytic system to prepare polygermanes in high yields (Scheme 5). In the reaction, element–element bonds are produced with the concurrent elimination of methane. With this rection, poly(trimethylgermane) (**6**) (*M*_n_ = 5,000, *M*_w_/*M*_n_ = 29, determined by viscometry) was obtained from trimethylgermane (Me_3_GeH) in 85% isolated yield.^[32]^ Because of branched microstructures, the polygermanes had extremely low intrinsic viscosities (0.06 dL/g). The catalytic production of either linear or branched polygermane accompanied by 1,3‐migration in the germyl–germylene complex results in the formation of hyperbranched polymers. In contrast, the use of dimethylphenylgermane (Me_2_GePhH) as a monomer limits the progress of the migration and poly(dimethylphenylgermane) (**7**) (*M*_n_ = 7900 and *M*_w_/*M*_n_ = 1.2) with a small polymer dispersity index was obtained.^[33]^

An electroreductive reaction is also studied as an effective synthetic method for polygermanes (Scheme 6). Through the electroreductive reaction with Pt cathode and Ag anode, polygermanes (R_1_, R_2_ = *n‐*butyl (^*n*^Bu) (**8**), *n*‐pentyl (^*n*^Pen) (**9**), *n*‐hexyl (^*n*^Hex) (**10**)) were obtained in moderate yields (20%–45%), high current efficiency (25%–50%), and moderate molecular weights (*M*_n_ = 2000–6,100, *M*_w_/*M*_n_ = 1.7–7.1).^[37]^ The obtained polygermanes also showed thermochromism similarity to the other polygermanes. With a Mg cathode and anode in a single compartment cell under ultrasound, poly(*n*‐butylphenylgermane) (**11**) (*M*_n_ = 19,900) was able to be prepared in 10% yield.^[39]^ As the features of the electrochemical reactions, the molecular weight and yield of polygermanes can be controlled by the amount of supplied electricity as well as the concentration of monomers.

It was found that polygermanes were synthesized by using a one‐electron reductant, samarium(II) iodide (SmI_2_), under mild homogeneous conditions (Scheme 7).[^41^, ^42^] Poly(diethylgermane) (**12**) (*M*_n_ = 4,330, *M*_w_/*M*_n_ = 1.13) with relatively low molecular weight and a narrow molecular‐weight distribution was afforded from dichlorodiethylgermane in 25% yield.^[42]^ The polymerization using SmI_2_ should be suitable for preparing controlled polygermanes with a narrow molecular‐weight distribution.

Since some organogermanium compounds can produce germanium radicals upon light irradiation, applications as a photoinitiator for polymer synthesis are proposed.^[69]^ The formation and recombination of radical and germylene species by light irradiation can be used for the synthesis of polygermanes. The scission of the Ge−C bond in the dibenzoyldiethylgermane (DBDEG) monomer and subsequent production of poly(diethylgermane) (**13**) (*M*_n_ = 2,750, *M*_w_/*M*_n_ = 1.11) proceeded by photoirradiation of visible light (440 nm, 0.14 × 10^−1^ (mW/cm^2^)) for 2.5 h (Scheme 8).^[43]^ The low‐molecular‐weight oligomers were produced by an increase in light intensity or reaction time. By using polymer **13** as a macrophotoinitiator, polystyrene with *M*_n_ = 8800 (5.0% conversion) was obtained from bulk styrene containing polymer **13** (1.3 × 10^−2^ M) by light irradiation (*λ* > 300 nm) for 1 h. The resulting polymer was polystyrene and no trace of polymer **13** was detected. In the absence of polymer **13**, polymerization of styrene did not proceed after the same irradiation time.

Recently, it was proposed that polygermanes can be prepared by a topotactic deintercalation of Ca ion from CaGe.^[44]^ Historically, the preparation of polydihydrogermane, (GeH_2_)_*n*_, from CaGe by using the topotactic deintercalation has been already suggested, while characterization data were not sufficient.[^70^, ^71^] (GeH_2_)_*n*_ (**14**) was prepared by two complementary 1‐ and 2‐step deintercalation methods (Method 1 and 2, respectively) (Scheme 9). In both cases, although polymer **14** comprised GeH_2_ repeating units, the product morphologies substantially differed. The aggregates of polymer **14** obtained by gentle 2‐step deintercalation had morphologies reminiscent of the aligned Ge_*n*_ chains, which were present in the CaGe precursor and were consistent with topotactic removal/replacement of Ca ions. From the X‐ray diffraction (XRD) data, it was suggested that these more ordered aggregates should possess a slightly larger interchain distance compared with polydihydrogermane prepared via rapid HCl‐mediated deintercalation. The molecular weight was estimated to be 37,000 for a 100 nm long (GeH_2_)_*n*_ chain obtained by applying the Ge−Ge−Ge end‐to‐end distance measured with XRD. Interestingly, polymer **14** was able to be functionalized by using thermally‐induced hydrogermylation reactions. The reaction of polymer **14** and 1‐dodecene produced poly(di‐*n*‐dodecylgermane) (**15**). The molecular weight of polymer **15** was calculated to be 500,000–700,000, which is relatively smaller than that of the estimated value from polymer **14** due to the increased solution compatibility and possible chain cleavage arising during sonication. The synthetic strategy offers the opportunity for various functional catenated polygermanes.

In addition to well‐known UV absorption properties, polygermanes can show luminescence (Figure 2). Poly(methyltrimethoxysilylphenylgermane) (**16**) was prepared by a Ru‐catalyzed demethanative coupling reaction.^[34]^ Polymer **16** showed fluorescence in THF and the maximum photoluminescence wavelength (*λ*_PL_) was 369 nm under excitation at 332 nm. The film of polymer **16** also exhibited broadened fluorescence at around 370 nm. The polymer containing germole units in the main‐chain can also be used for luminescent materials. It was reported that poly(tetraphenyl)germole (**17**) exhibited the emission band with the peak at *λ*_PL_ = 499 nm.^[45]^ The emission was quenched by addition of nitroaromatics such as picric acid, 2,4,6‐trinitrotoluene (TNT), 2,4‐dinitrotoluene (DNT), and nitrobenzene. From these data, an application to the detection of explosives is proposed. As another example, the emission from poly(dithienogermole‐1,4‐diyl) (**18**) was investigated.^[28]^ The polymer showed blue emission at *λ*_PL_ = 431 nm with 5% of the absolute photoluminescence quantum yield (*Φ*_PL_). The value of *λ*_PL_ was longer than that of monomeric and dimeric derivatives, implying that electronic communication might be constructed through polymer main‐chains. The similar type of the polygermane with cyclic substituent can be used for a photopatterning material.^[46]^ A poly(cyclogermapentene) (**19**) was prepared by the metal‐catalyzed dehydrogenative coupling reaction, and exposure of the resulting polygermanes to UV light led to elimination of organobutadiene from the polymer side‐chains and deposition of germanium metal. This method proposes an efficient method to fabricate patterns with semiconducting Ge under mild conditions for optoelectronic applications.

The optical properties of polygermanes are evaluated by quantum chemical calculations with density functional theory (DFT). According to the results of the calculations, the bandgaps of polygermanes can be modulated by the side‐chains. For instance, the bandgap energies of (GeH_2_)_*n*_, (GeHPh)_*n*_, and (GePh_2_)_*n*_ are estimated to be 3.03, 2.72, and 2.13 eV, respectively.^[52]^ From the simulated charge density distribution and partial density of states of Ge atoms, the lowest unoccupied molecular orbital (LUMO) is dominantly constructed by the Ge 4s and 4p_x_ atomic orbitals, while the highest occupied molecular orbital (HOMO) is mainly composed of the Ge 4p_z_ atomic orbital along the skeletal axis. The similar bandgap modulation is also shown in polysilanes and polystannanes. In the case of the polygermanes, the calculated average Ge–Ge bond lengths of (GeH_2_)_*n*_, (GeHPh)_*n*_, and (GePh_2_)_*n*_ are 2.451, 2.469, and 2.549 Å, respectively. This result indicates that the elongation of the Ge–Ge bond likely contributes to the decrease in the bandgap energies. Interestingly, it is predicted that the bandgap of (GeHPh)_*n*_ can be reduced to 1.131 eV by 16% tensile strain, which elongated the Ge–Ge bond. As the tensile strain increases, the HOMO band exhibits little changes, while the LUMO band approaches the Fermi level, leading to the reduction of the bandgaps. Under the large tensile strain, the HOMO state still originates from the Ge 4p_z_ atomic orbitals, whereas the LUMO state is mainly contributed by the side‐chain substituents. It is also theoretically proposed that the degree of electron delocalization along the polymer main‐chain depends on the conformation of the main‐chain and the catenated group 14 elements.^[12]^ From these considerations, polygermanes have the potential to be optical functional materials with tunable bandgap energies.

Not only the properties of the 1D polygermanes but also the properties of a 2D germanium sheet of (GeH)_*n*_ are calculated with DFT.^[72]^ In this research, it is theoretically suggested that the germanium sheet polymer should have a direct bandgap of 1.7 eV, whereas the silicon analog (SiH)_*n*_ should have the indirect bandgap of 2.7 eV. Furthermore, the germanium sheet polymer showed luminescence with significantly smaller Stokes shift compared to the luminescent silicon analog called siloxene (SiHSiOH)_*n*_. This should be because of the missing chemical disorder of the OH groups, which are randomly bonded to the back sheet consisting of the {111} double layers in siloxene but are absent in the germanium sheet polymer.

#### 2.1.2 Germanium‐containing polymers in the main‐chain

**SCHEME 10:**
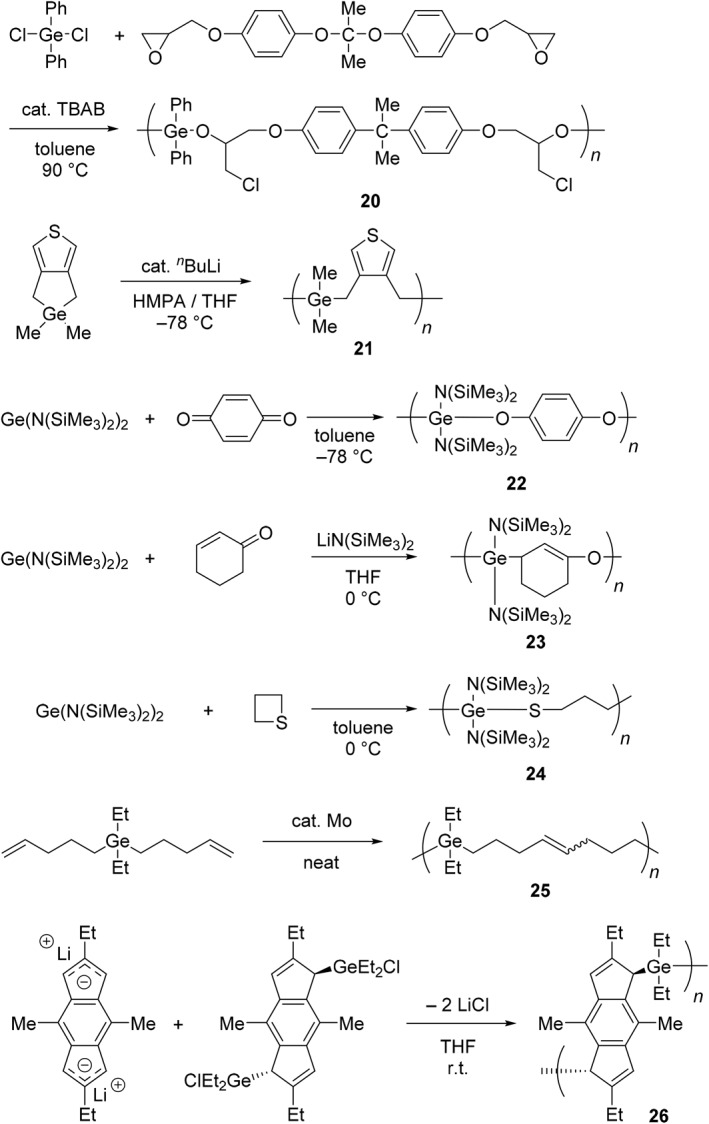
Synthetic methods of germanium‐containing polymers in the main‐chain (**20**–**26**).

**SCHEME 11:**
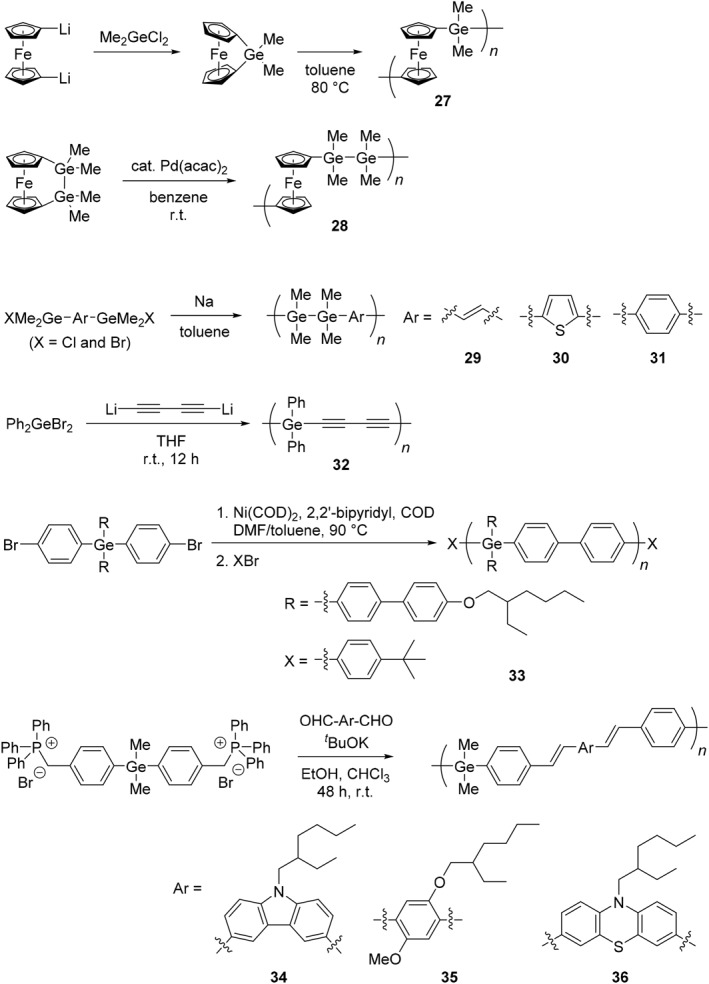
Synthetic methods of germanium‐containing polymers in the main‐chains (**27**–**36**).

**SCHEME 12:**
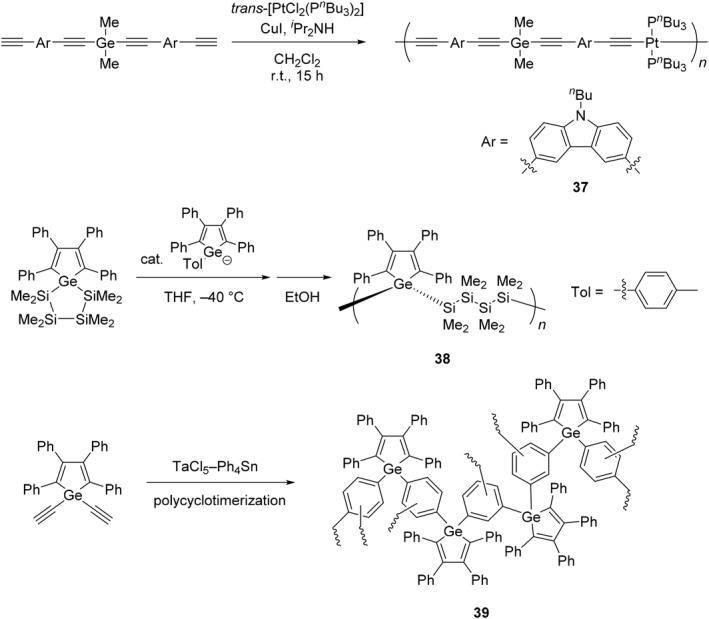
Synthetic methods of germanium‐containing polymers in the main‐chains (**37**–**39**).

Focusing on the unique reactivity of organogermanium compounds, various types of polymers containing germanium in the main‐chain have been synthesized, and the functional properties originating from the heavy element have been explored (Schemes 10, 11, 12 and Table 2). Poly(germyl ether) (**20**) was prepared by polyaddition of dichlorodiphenylgermane and bisphenol A diglycidyl ether (Scheme 10).^[73]^ The reaction proceeded smoothly and regioselectively at high temperature (90°C) in the presence of a catalytic amount of tetrabutylammonium salt (TBAB, 5 mol%). While the resulting poly(germyl ether) was stable in the solid state, it was more sensitive to water than its silicon analog.^[90]^ The reactivity of poly(germyl ether) can be used to induce a gradual decomposition in the solution.

**TABLE 2:** Data and properties of germanium‐containing polymers in the main‐chain.

	Polymer type	Synthetic method	*M*_n_	*M*_w_	Properties	References
**20**	Polyether	Polyaddition	8000	12,200	Stable in the solid state Water sensitive in solution	[73]
**21**	Thiophene copolymer	Ring‐opening polymerization	47,500	96,500	Light absorption (*λ*_abs_ = 269 nm) Thermally stable up to 260°C	[74]
**22**	Polyether	Redox copolymerization	55,000	130,000	−	[75]
**23**	Polyenolate	Michael‐type addition	130,000	420,000	Transparent film by casting	[76]
**24**	Polythioether	Ring‐opening polymerization	52,800	114,000	−	[77]
**25**	Vinylene copolymer	Acyclic diene metathesis polymerization	14,800	26,500	Viscous liquid Metal Ge after thermal decomposition	[78]
**26**	*s*‐Indacene copolymer	Transmetallation	3700	8000	Thermally stable up to 350°C	[79]
**27**	Polymetallocene	Ring‐opening polymerization	161,900	356,200	High refractive index film	[80, 81]
**28**	Polymetallocene	Palladium or platinum catalytic system	240,000	400,000	High refractive index film	[82]
**29**	Vinylene copolymer	Wurtz‐type coupling	−	33,000 (peak)	Electrical conductivity	[83]
**30**	Thiophene copolymer	Wurtz‐type coupling	11,000	27,500		
**31**	Phenylene copolymer	Wurtz‐type coupling	7800	21,300		
**32**	Dialkyne copolymer	Transmetallation	1683	2255	Electrical conductivity	[84]
**33**	Biphenyl copolymer	Yamamoto coupling reaction	42,000	110,000	High triplet energy Charge mobility	[85]
**34**	Arylene copolymer	Wittig‐condensation polymerization	2600	6000	Luminescence white for **34** (*λ*_EL_ = 488, 663 nm)	[86]
**35**	Arylene copolymer	Wittig‐condensation polymerization	2300	6200	Yellowish green for **35** (*λ*_EL_ = 503 nm)	
**36**	Arylene copolymer	Wittig‐condensation polymerization	3100	8100	Yellow for **36** (*λ*_EL_ = 536 nm)	
**37**	Pt‐containing copolymer	Dehydrohalogenating‐type reaction	14,250	15,240	Phosphorescence (*λ*_Phos_ = 460 nm, *Φ*_Phos_ = 22.6%)	[87]
**38**	Germole‐containing polymer	Ring‐opening polymerization	12,000	19,000	Light absorption (*λ*_abs_ = 320 nm) Luminescence (*λ*_PL_ = 520 nm)	[88]
**39**	Germole‐containing polymer	Polycyclotrimerization	2900	5160	Light absorption (*λ*_abs_ = 505 nm) Luminescence (*λ*_PL_ = 540 nm)	[89]

Anionic ROP of cyclic organogermanium compounds can be conducted by using a nucleophilic initiator. The polymer (**21**) containing thiophene and germanium units in the main‐chain was obtained by the reaction of 1‐germa‐1,1‐dimethyl[3,4,c]thienocyclopentane in the presence of *n*‐butyllithium and hexamethylphosphoric triamide (HMPA) catalysts in THF.^[74]^ The mechanism was postulated to be that *n*‐butyllithium first binds to the germanium center to form an anionic higher coordinated state, followed by ring opening to provide an anionic methylene. The generated methylene anion attacks another cyclic germanium monomer, and this reaction proceeds continuously to afford the polymer product. The melting point and glass transition temperature of polymer **21** were measured to be 105–107°C and −11.0°C, respectively. Polymer **21** was thermally stable up to 260°C in an inert atmosphere. In the UV absorption spectrum, it was found that polymer **21** had a shoulder peak at 269.0 nm, which was not detected in the monomer (*λ*_abs_ = 258.0 nm). The bathochromic‐shifted shoulder peak should be attributed to the through‐space π–π interactions between neighboring non‐conjugated thiophene rings.

The alternating copolymers including germanium units can be prepared by an oxidation–reduction (redox) copolymerization.[^75^, ^91^] For example, it was reported that the copolymerization of bis[bis(trimethylsilyl)amido]germanium (germylene precursor) and *p*‐benzoquinone as smoothly proceeded at −78°C in toluene without any addition of a catalyst.^[75]^ Consequently, the corresponding copolymer (**22**) was obtained in a high yield (85%) within 1 h. Copolymer **22** has alternating germanium and *p*‐hydroquinone units in the main‐chain and is soluble in common organic solvents. At the initial step, the formation of zwitterion or diradical should be induced by the reaction with germylene (reductant monomer) and *p*‐benzoquinone (oxidant monomer).

From the same germylene precursor, poly (germanium enolate) can also be synthesized with a cyclic *α*,*β*‐unsaturated ketone in the presence of a catalytic amount of an organolithium reagent.^[76]^ The copolymerization by Michel‐type addition was carried out at 0°C in THF, and the resulting copolymer (**23**) was isolated as a white powder. The transparent film of polymer **23** was able to be prepared by a casting method owing to good film‐formability originating from the enough molecular weight (*M*_n_ = 130,000, *M*_w_/*M*_n_ = 2.16). A glass transition temperature and a melting point of polymer **23** were determined to be 40.2°C and 220.8°C, respectively. It is well known that decomposition of Ge−N and Ge−O bonds in the enolate moiety is easily caused by the attack of nucleophiles such as alcohol or water. On the other hand, polymer **23** is highly resistant toward potential reactive species. This is probably because the steric hindrance of the bulky trimethylsilyl group might prevent water molecules from attacking the germanium atoms. The germylene derivatives can also be used for alternating ring‐opening copolymerization with thietane. The reaction was conducted in toluene at 0°C to provide moderate molecular‐weight poly (germanium thiolate) (**24**).^[77]^ The polymer has unique Ge−S bonds in the main‐chain.

Unsaturated polycarbogermanes were prepared by acyclic diene metathesis (ADMET) polymerization with germanadiene monomers in the presence of molybdenum, ruthenium, or tungsten catalysts.[^78^, ^92^] For instance, the polymer (**25**) was obtained as a viscous liquid with a glass transition temperature at −89°C.^[78]^ From the result of TGA, 17% weight of polymer **25** was recovered after heating to 800°C under an N_2_ atmosphere. Although the metal recovery is not quantitative (e.g., the expected metal content in polymer **25** is ca. 29%), the result indicates that germanium‐containing polymers have potential applications in the production of elemental germanium.

By using a condensation reaction between bis‐chlorogermyl‐*s*‐indacene and *s*‐indacenyl lithium derivatives, the copolymers involving unique steric structures were synthesized.^[79]^ The resulting poly(*s*‐indacenylgermane) (**26**) has coplanar tricyclic rings and two germanium atoms projected at opposite (*trans*) directions from the π‐surface. Polymer **26** was thermally stable up to 350°C, which is higher than its polymer derivative connected by carbodiimides, poly(germyl‐*s*‐indacene carbodiimides) (stable up to 250°C).

Polymetallocenes are a type of inorganic polymers composed of an alternating ferrocene unit and a main group element having aryl or alkyl groups (Scheme 11).[^80^, ^81^, ^82^, ^93^, ^94^, ^95^, ^96^] ROP techniques are applicable to the synthesis of polymetallocenes. Since polymetallocenes contain many inorganic elements, it is expected that the materials with high refractive indices (*n*) should be obtained by incorporating heavy elements in their polymer backbones. It was reported that the polyferrocenylgermanes were able to be prepared by a thermal ROP of germa[1]ferrocenophanes in toluene at 80°C.^[80]^ For instance, the resulting polymer (**27**) had enough molecular weight to form a polymer film, and the film showed a high refractive index (*n* = 1.689) at 589 nm.^[81]^ The reaction with a palladium or platinum catalyst is also effective in the preparation of polyferrocenylgermanes.[^82^, ^95^] With this reaction, the polymer containing Ge–Ge bonds (**28**) were synthesized from 1,2‐digerma[2]ferrocenophane at room temperature.^[82]^ This polymer has the potential to be applied as a high‐refractive‐index film or thin microlens because it contains heavier elements.

Organogermanium polymers with σ‐ or σ–π‐conjugation exhibit unique physical properties derived from the electronic state of the conjugated system and have attracted attention for their use as semiconductors.[^97^, ^98^] The conductivities of germanium‐containing polymers were investigated by preparing the polymers composed of Ge−Ge bonds connected by three types of π‐conjugated linkers, vinylene (**29**), thiophene (**30**), and phenylene (**31**).^[83]^ After antimony pentafluoride (SbF_5_) doping, the polymers showed electrical conductivities of the order of 10^−4^ S/cm, which are similar to or a little higher than those of corresponding silicon analogs. Similar conductivity (10^−4^ S/cm) was obtained in the case of germanium‐containing polymer (**32**) with dialkyne linkages after doping with iron(III) chloride (FeCl_3_).^[84]^ These results suggest that organogermanium polymers with σ–π‐conjugation should be promising candidates for semiconductor materials.

It was found that significant differences in triplet energy (*E*_T_) of σ–π‐conjugated polymers were observed depending on the incorporated group 14 elements.[^85^, ^99^, ^100^] The germanium‐containing polymer (**33**) with σ–π‐conjugation was prepared through a Yamamoto coupling reaction from the corresponding dibrominated germanium monomer.^[85]^ The device performances were altered by the types of the including elements (C, Si, Ge, and Sn), and polymer **33** showed the best performance as a host material owing to its higher *E*_T_ (2.83 eV) and reasonable charge mobility (10^−8^ to 10^−7^ cm^2^/(V · s)). It is implied that the discovery of various optoelectronic properties depending on the elements should be potential guidelines for tuning the electronic states of π‐conjugated polymers.

By using Wittig‐condensation polymerization, poly(*p*‐phenylenevinylene) (PPV) derivatives containing germanium can be prepared, and their light‐emitting properties were explored. The three‐types of copolymers with carbazole (**34**), dialkoxybenzene (**35**), and phenothiazine (**36**) moieties were synthesized.^[86]^ Accordingly, polymers **35** and **36** exhibited yellowish green (*λ*_EL_ = 503 nm) and yellow (*λ*_EL_ = 536 nm) electroluminescence (EL), respectively. Interestingly, polymer **34** showed white electroluminescence (*λ*_EL_ = 488, 663 nm) with a CIE coordinate of (0.33, 0.37) derived from electroplex emission in the light‐emitting diode (LED). Their decomposition temperatures with 5% weight losses of the polymers were measured to be 404°C for **34**, 387°C for **35**, and 389°C for **36**, indicating sufficient thermal stability for OLED applications.

As another example of luminescent polymers, phosphorescence was observed from the germanium‐bridged platinum metallopolyynes. The polymer (**37**) was synthesized by a classical dehydrohalogenating‐type reaction with *trans*‐[PtCl_2_(PBu_3_)_2_] and germanium‐containing terminal dialkyne in the presence of an amine base and cupper iodide (Scheme 12).^[87]^ Polymer **37** showed weak luminescence (*Φ*_PL_ = 0.62%, *τ* = 0.98 ns) around room temperature (290 K), whereas it exhibited the strong phosphorescence (*Φ*_Phos_ = 22.6%, *τ* = 8.23 μs) at frozen temperature (20 K). The phosphorescence efficiency of polymer **37** was higher than that of the silicon analog (*Φ*_Phos_ = 15.8%, *τ* = 2.43 μs) owing to the internal heavy‐atom effect of germanium.

The germole‐containing polymer (**38**) was synthesized by ROP with tetraphenylgermole‐*spiro*‐cyclogermatetrasilane.^[88]^ Interestingly, the polymerization mechanism is different from the silole analog, in which the silole forms five coordinated intermediates by the attack of nucleophile. In contrast, the germole monomer forms germole anion by the attack of nucleophile, and the subsequent propagation step proceeds. The resulting polymer **38** showed characteristic UV absorption and emission bands with the peaks at *λ*_abs_ = 320 nm and *λ*_PL_ = 520 nm, respectively.

The germole‐contaning hyperbranched polymer (**39**) was prepared through copolycyclotrimerization of terminal alkyne moieties.^[89]^ The polymer structure was obtained as the mixture of 1,2,4‐ and 1,3,5‐conformers of benzene moieties. Despite the all‐aromatic nature of polymer **39**, the product is completely soluble in common organic solvents owing to its highly branched molecular structure. The weight‐average molecular weight of the hyperbranched polymer **39** was estimated to be *M*_w_ = 5160 (*M*_w_/*M*_n_ = 1.78), and the absorption and emission bands were observed at around 505 and 540 nm, respectively. In addition, polymer **39** had high thermal stability up to 350°C.

#### 2.1.3 Germanium‐containing π‐conjugated polymers

**FIGURE 3:**
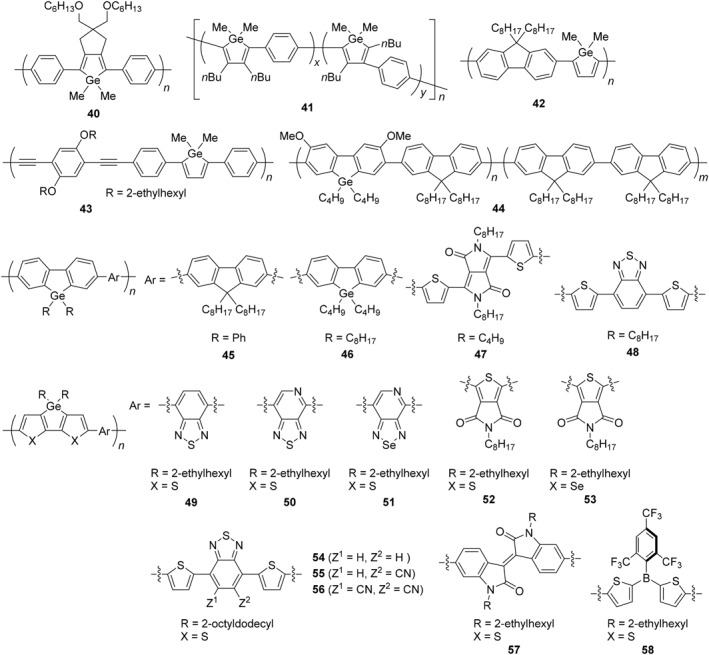
Structures of germanium‐containing π‐conjugated polymers (**40**–**58**).

**FIGURE 4:**
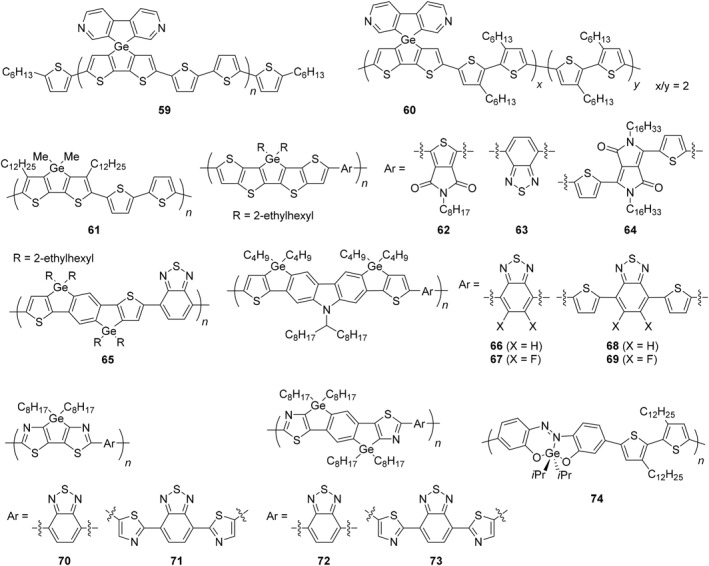
Structures of germanium‐containing π‐conjugated polymers (**59**–**74**).

π‐Conjugated polymers are famous as a typical example of polymers applicable to light‐emitting and conductive materials.^[101]^ The structures are basically composed of *sp* ^2^ and/or *sp* carbons, and benzene rings are often used to construct π‐conjugated systems because of their high stability and ease of chemical modification. For synthesis of π‐conjugated polymers, cross‐coupling reactions with transition metals are effective in making the connection between *sp* ^2^ and/or *sp* carbons.^[102]^ The electronic properties of the π‐conjugated system can be modulated by incorporating heteroatoms,[^103^, ^104^, ^105^, ^106^] and germanium has also been introduced into π‐conjugated polymer backbones to control their energy levels of frontier molecular orbitals (FMOs), HOMO and LUMO (Figures 3, 4 and Table 3).^[129]^

**TABLE 3:** Optical data and applications of germanium‐containing π‐conjugated polymers.

	*M*_n_	*M*_w_	*λ*_abs_/nm	*λ*_PL_/nm	*Φ*_PL_/%	Application	References
**40**	20,000	58,000	442	500	79	−	[107]
**41**	3400	7200	420 (onset)	−	−	−	[108]
**42**	4400	7500	465	547	4	Chemosensor	[109]
**43**	3870	8510	ca. 427	471	14	−	[110]
**44**	21,800	34,900	388	438 (film)	70 (film)	Organic light‐emitting diode	[111]
**45**	20,000	56,000	383	429	22	Laser emission	[112]
**46**	10,000	19,000	ca. 383	415	54	−	[113]
**47**	13,000	37,000	688 (film)	−	−	Solar cell	
**48**	10,000	24,000	580 (film)	−	−	(PCE: 1.5% for **47**) (PCE: 2.8% for **48**)	
**49**	12,400	21,100	691	−	−	Solar cell (PCE: 2.9%)	[114]
**50**	6200	9300	745	−	−	Solar cell	[115]
**51**	5700	8550	741	−	−	(PCE: 3.46% for **50**) (PCE: 0.18% for **51**)	
**52**	48,000	82,000	679 (film)	−	−	Solar cell (PCE: 7.3%)	[116]
**53**	41,000	62,000	728 (film)	−	−	Solar cell (PCE: 5.2%)	[117]
**54**	39,000	97,500	676 (film)	−	−	Solar cell	[118]
**55**	42,000	109,200	786 (film)	−	−	(PCE: 3.51% for **54**)	
**56**	20,500	44,400	833 (film)	−	−	(PCE: 6.55% for **55**) (PCE: 0.63% for **56**)	
**57**	59,000	148,000	742 (film)	−	−	Ambipolar charge transport	[119]
**58**	15,300	55,100	550, 517	630	36	−	[120]
**59**	850	1090	489	575	32	−	[121]
**60**	5530	17,900	406	549	12		
**61**	41,000	68,000	ca. 550 (film)	−	−	−	[122]
**62**	12,000	17,000	595, 643	−	−	Solar cell (PCE: 7.16%)	[123]
**63**	10,000	35,000	669, 712	−	−	Solar cell	[124]
**64**	32,000	64,000	478, 692	−	−	(PCE: 4.66% for **63**) (PCE: 0.87% for **64**)	
**65**	37,000	48,000	645 (film)	−	−	Solar cell (PCE: 6.5%)	[125]
**66**	55,200	98,300	604 (film)	−	−	Solar cell	[126]
**67**	22,000	29,700	587 (film)	−	−	(PCE: 3.03% for **66**)	
**68**	26,800	45,600	608 (film)	−	−	(PCE: 1.96% for **67**)	
**69**	17,900	31,000	572 (film)	−	−	(PCE: 1.63% for **68**) (PCE: 4.05% for **69**)	
**70**	8800	22,000	630 (film)	ca. 710 (toluene)	−	−	[127]
**71**	3100	4900	555 (film)	ca. 650 (toluene)	−		
**72**	7700	12,000	608 (film)	650 (toluene)	−		
**73**	8700	25,000	577 (film)	ca. 685 (toluene)	−		
**74**	14,000	36,000	693	770	10	−	[128]

Germole is a class of metaloles (1‐heterocyclopenta‐2,4‐dienes) with a germanium atom introduced at the 1 position in the five‐membered ring. The LUMO energy level of germole is lower than that of the carbon analog (cyclopentadiene) because of σ*–π* conjugation between σ* orbital of the germanium moiety and π* orbital of the carbon‐based π‐conjugated system.^[14]^ The strength of the σ*–π* conjugation is predicted to increase in the order silole (Si) < germole (Ge) < stannole (Sn). On the other hand, since the bond distances between carbon and heavy elements also increase in the same order, the orbital overlap efficiency decreases in the order silole (Si) > germole (Ge) > stannole (Sn). Consequently, silole, germole, and stannole have similar LUMO energy levels.^[14]^ In π‐conjugated polymers, it is theoretically suggested that silole units work as a quinoid unit rather than an aromatic unit because of the small resonance energy of the silole ring without lone‐pair electrons to form the 6π‐electron system.[^130^, ^131^] Therefore, the π‐electron can be efficiently delocalized throughout the polymer backbone, resulting in a narrower energy gap. This feature should be common to the heterole units with group 14 elements. From these backgrounds, numerous germole compounds have been synthesized and their properties have been investigated because elemental replacements allow fine tuning of the properties without changing the structure of the framework.

As the example of the germole‐containing polymer, the polymer (**40**) was synthesized by a Yamamoto coupling reaction with dihalogermoles (Figure 3).^[107]^ Polymer **40** exhibited strong emission in the visible region (*λ*_PL_ = 500 nm, *Φ*_PL_ = 79%) even though the repeating unit showed weaker emission in the shorter wavelength region (*λ*_PL_ = 452 nm, *Φ*_PL_ = 1.8%). Synthesis of π‐conjugated polymers containing germole moieties in the main‐chain (**41**) was also achieved by the post‐element‐transformation technique from the corresponding organotitanium polymers.^[108]^ The polymer had broadened absorption bands and the onset value in the long wavelength region reached 420 nm. Interestingly, the polymer had a low‐lying LUMO energy level (−3.95 eV). These properties suggest that this polymer could be potentially applicable to advanced materials such as electron‐transporting organic semiconductors. The alternating copolymer containing germole and fluorene units (**42**) was also prepared by the post‐element transformation method in which tellurophene was converted to germole through di‐lithium intermediate.^[109]^ Polymer **42** showed greenish emission (*λ*_PL_ = 547 nm, *Φ*_PL_ = 4%). The LUMO energy level of polymer **42** (−3.65 eV) was lower than that of the corresponding group 16 element‐containing polymer (thiophene, −3.16 eV) and almost comparable to that of the group 15 element‐containing polymer (phosphole, −3.60 eV). As an application, polymer **42** can be used as a chemosensor. It was demonstrated that polymer **42** proceeded acidolysis at the germole moiety to 1,4‐diphenyl‐1,3‐butadiene in the HCl/THF solution accompanied by blue‐shifted emission colors from green to blue. Polymer **42** also worked as a fluoride ion chemosensor with spectral changes in UV–vis absorption originating from the formation of hypervalently coordinated species at germanium. The polymer containing phenylene‐ethynylene moieties (**43**) was also prepared by the same method, and polymer **43** showed more efficient luminescence in the visible region (*λ*_PL_ = 471 nm, *Φ*_PL_ = 12%).^[110]^

Germafluorene is one of the stable heterofluorenes, and the LUMO energy level is different from fluorenes or silafluorene because of σ*–π* conjugation effect involving germanium. The first germafluorene‐containing polymer (**44**) was synthesized as the π‐conjugated copolymer with fluorene having 10% of germafluorene units in the polymer backbone.^[111]^ Polymer **44** showed blue emission in the slightly shorter wavelength region than polyfluorene, and was applied to its single layer EL device exhibiting high brightness of 2630 cd/m^2^ at 7.8 V. The alternating copolymer (**45**) composed of germafluorene and fluorene units was prepared, and the emission wavelength was similar to polyfluorene.^[112]^ Interestingly, the emission efficiency of polymer **45** was slightly higher (*Φ*_PL_ = 22%) than that of the polyfluorene analog (*Φ*_PL_ = 17%). The synthesis and evaluation of homopolymers and alternating donor–acceptor (D–A) copolymers consisting of different aromatic comonomers have been carried out to reveal their optical properties and photovoltaic performances.^[113]^ The homopolymer (**46**) and D–A copolymers (**47** and **48**) were air‐stable and had optical bandgaps (*E*_g_) of 2.95 eV for **46**, 1.63 eV for **47**, and 1.79 eV for **48**. The bandgap of polymer **46** was comparable to the polyfluorene and polysilafluorene analogs (*E*_g_ = 2.93 eV), and polymer **46** also exhibited emission at 415 nm with a high quantum yield (*Φ*_PL_ = 54%). Because of their low bandgap properties, polymers **47** and **48** were tested for their performances in field‐effect transistors (FETs) and bulk heterojunction (BHJ) photovoltaic cells. Consequently, polymer **47** showed the best results in FETs with a hole mobility up to 0.04 cm^2^/(V·s). In terms of photovoltaic applications, polymer **48** recorded a power conversion efficiency (PCE) of 2.8%.

Dithienogermole can be used as an electron‐donating unit to modulate the energy levels of π‐conjugated polymers. Low bandgap polymer (**49**) was synthesized by constructing a D–A π‐conjugated system between the dithienogermole donor and the benzothiadiazole acceptor. Polymer **49** showed strong absorption band at 691 nm, and the polymer film was able to be applied for photovoltaic cell with PCE of 2.9%.^[114]^ By converting the acceptor commoner to the more electron deficient one, further improvement of the performance for photovoltaic cell was expected. The alternating copolymers composed of the dithienogermole donor and the pyridinothiadiazole or selenadiazole acceptor (**50** and **51**) were prepared.^[115]^ Polymers **50** and **51** exhibited absorption bands at 745 and 741 nm, respectively. The PCE of **50** and **51** in BHJ‐type polymer solar cells as blends with PC_70_BM were 3.46% and 0.18%. The PCE of **51** was low, however the spectral edges reached approximately 960 nm, reflecting the low‐bandgap properties. D–A type copolymer (**52**) with *N*‐octylthienopyrrolodione had the absorption band at 679 nm with the low bandgap (*E*_g_ = 1.69 eV) and a higher HOMO level than the dithienosilole analog (*λ*_abs_ = 670 nm, *E*_g_ = 1.73 eV).^[116]^ In the BHJ solar cell, polymer **52** displayed an average PCE of 7.3% when used in simple inverted device architectures without interlayers. The similar copolymer (**53**) with diselenogermole was also prepared, and it showed the absorption band at longer wavelength region (*λ*_abs_ = 728 nm) with the low bandgap (*E*_g_ = 1.60 eV) and the PCE of 5.2%.^[117]^ In the case of the dithienogermole copolymers (**54**–**56**) with the 4,7‐bis(thiophen‐2‐yl)‐2,1,3‐benzothiadiazole derivative as electron‐acceptors, the maximum PCE (6.5%) was obtained from monocyano substituted polymer (**55**).^[118]^ The decrease in the LUMO energy level from non‐cyano polymer **54** (−3.27 eV) to monocyano polymer **55** (−3.54 eV) leads to an improvement in the light‐harvesting properties of the device without compromising the dissociation yield of the polymer exciton owing to the optimized the position of the polymer LUMO level closer to that of the electron acceptor (6,6)‐phenyl C_71_ butyric acid methyl ester (PC_71_BM, −3.91 eV). On the other hand, di‐cyano polymer **56** showed poor device performance (PCE: 0.63%) despite a much closer LUMO energy level (−3.77 eV) to PC_71_BM. This is probably due to the poor orbital energy level alignment with PC_71_BM. By connecting dithienogermole units with isoindigo‐based units, polymer **57** showed the lowest energy gap (1.50 eV) and LUMO energy level (−3.98 eV) among the isoindigo‐based polymer analogs.^[119]^ In addition, polymer **57** had an ambipolar charge transport property in FETs. Effective D–A interactions can be constructed by combining unique electron‐deficient comonomers, tricoordinate borane moieties.^[120]^ The resulting π‐conjugated polymer (**58**) exhibited solvatochromic effects in the fluorescence spectra from 630 nm (in hexane, *Φ*_PL_ = 36%) to 670 nm (in pyridine, *Φ*_PL_ = 4%), indicating a distinct intramolecular charge transfer (ICT) character in the excited state.

Various π‐conjugated units based on dithienogermole scaffolds have been developed not only because of their excellent functionality but also because of their great molecular designability and extensibility. In the spiro‐fused structure composed of dithienogermole and dipyridinogermole units, it was suggested that the emission efficiency was reduced by intramolecular photoinduced energy transfer and electron transfer from the dipyridinogermole to dithienogermole moieties.^[121]^ The relatively planar structure of polymer **59** contributed to an increase in the emission efficiency (*Φ*_PL_ = 32%) owing to extension of the main‐chain π‐conjugation, whereas the relatively twisted structure of polymer **60** resulted in a decrease in the emission efficiency (*Φ*_PL_ = 12%).

The molecular planarity can be affected by the atom sizes of germanium and silicon. For instance, the singlet–triplet intersystem crossing rate of the more planar dithienogermole‐containing polymer (**61**) was smaller than that of the silicon analog beyond the heavy‐atom effect.^[122]^ It has been proposed that highly ring‐fused structures are advantageous for improving the performance of organic solar cells. The coplanarity of the π‐conjugated polymers was enhanced by using dithienogermolodithiophene units (**62**–**64**).[^123^, ^124^] The BHJ solar cell with PC_71_BM blend afforded the PCE up to 7.2% for polymer **62** without thermal annealing or processing additives. The D–A polymer (**65**) having germanium‐bridged indacenodithiophene and benzothiadiazole as donor and acceptor units, respectively, was highly soluble and showed the PCE of 5.0% with the PC_71_BM blend.^[125]^ By using dithienogermolocarbazole as a donor, the D–A polymers (**66**–**69**) containing benzothiadiazole acceptors with/without thiophene linkers were synthesized.^[126]^ It was found that the extra fluorine atoms on the benzothiadiazole unit reduced the HOMO energy level while the LUMO energy level remained less affected. Additionally, the fluorinated copolymers exhibited higher mobilities than the corresponding non‐fluorinated counterparts. Among the polymers, polymer **69** exhibited the best PCE (4.05%) with a good hole mobility (3.97 × 10^−3^ cm^2^/(V · s)). In the case of thiazole‐containing copolymers (**70**–**73**), they had lower HOMO and LUMO energy levels than thiophene‐based analogs, and those polymers are potentially applied to active layers in organic thin film transistors.^[127]^

It is known that heavy main‐group elements have room to formally possess more than eight electrons around the elements beyond the Lewis octet rule without using *d* orbital.^[132]^ The excess electrons are placed away from the central element by forming three‐center four‐electron (3c‐4e) bonds, and it is considered that the situation satisfies the Lewis octet rule and the compound can exist stably. Such compounds are called as hypervalent compounds,^[133]^ and they show unique optical properties originating from the polarized 3c‐4e bonds.[^134^, ^135^, ^136^, ^137^] It was discovered that the π‐conjugated polymer (**74**) containing the hypervalent germanium moiety in the main‐chain exhibited highly excellent near‐infrared (NIR) absorption and emission both in solution (*λ*_abs_ = 693 nm, *λ*_PL_ = 770 nm, *Φ*_PL_ = 10%) and film (*λ*_abs_ = 710 nm, *λ*_PL_ = 807 nm, *Φ*_PL_ = 4%).^[128]^ The hypervalent germanium compounds with tridentate azobenzene ligands formed distorted trigonal bipyramidal geometry, in which Ge–N coordination at the equatorial position strongly reduced the LUMO energy level and the 3c‐4e bond in the apical position effectively elevated the HOMO energy level. Consequently, the hypervalent germanium compounds showed moderate NIR emission even in the small molecules.[^128^, ^138^] In polymer **74**, the germanium‐fused azobenzene moiety worked as a strong acceptor with the low‐lying LUMO energy level (−3.66 eV). From these results, it is suggested that the combination of hypervalent bonds and π‐conjugated systems is an effective strategy for the creation of NIR absorption and emission materials.

### 2.2 Sn‐containing polymers

#### 2.2.1 Polystannanes

**SCHEME 13:**
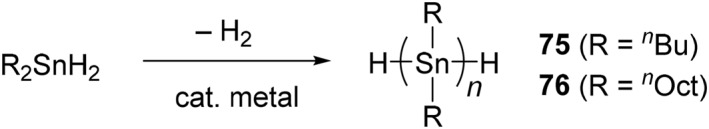
Synthesis of polystannanes by metal‐catalyzed dehydropolymerization.

**SCHEME 14:**
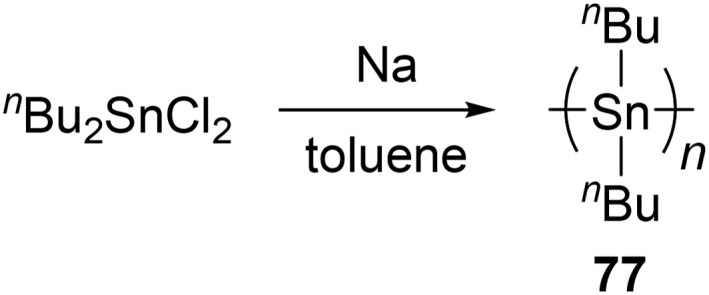
Synthesis of polystannanes by Wurtz‐type coupling.

**SCHEME 15:**
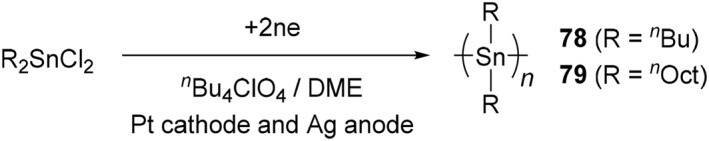
Synthesis of polystannanes by electroreductive synthesis.

**SCHEME 16:**
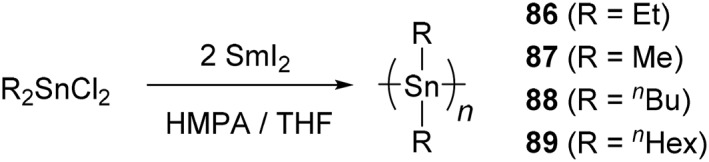
Synthesis of polystannanes by one‐electron reduction.

**SCHEME 17:**
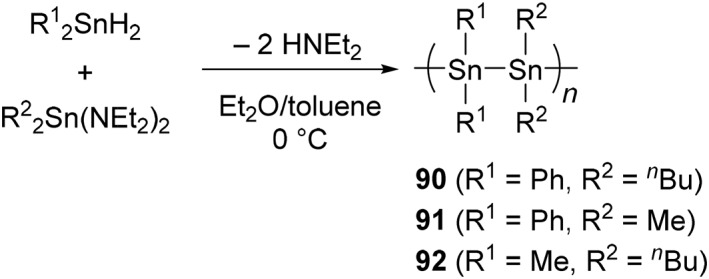
Synthesis of polystannanes by polycondensation.

**FIGURE 5:**
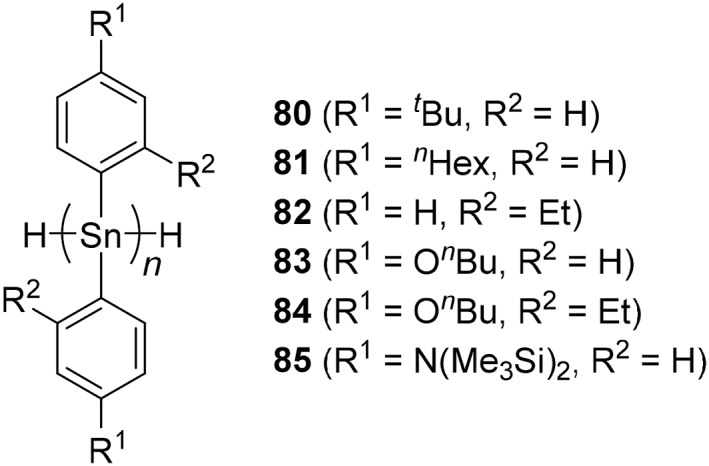
Structures of polystannanes synthesized by dehydropolymerization.

Polystannanes can be regarded as the only polymer with a backbone of covalently bonded metal atoms, and show unique properties among organometallic polymers.[^9^, ^139^] Tin is a heavier element than germanium, and the existence of polystannanes is known to be older than polygermanes. The synthesis of polystannane, poly(diethylstannane), has been already done in 1852.^[140]^ However, high molecular‐weight polystannanes (*M*_n_ > 10^6^) have not been reported until 1996.^[141]^ This is because that the high molecular‐weight polystannanes are more easily degraded by light and moisture into low molecular weight fractions compared to the polygermanes. In other words, careful synthesis and purification are required to obtain the high molecular‐weight polystannanes. Several reactions have been proposed for the preparation of oligo or polystannanes as well as the synthesis of polygermanes, such as a metal‐catalyzed dehydropolymerization,[^142^, ^143^, ^144^, ^145^, ^146^, ^147^, ^148^, ^149^, ^150^, ^151^, ^152^, ^153^, ^154^, ^155^, ^156^, ^157^, ^158^] a Wurtz‐type coupling,[^27^, ^141^, ^159^] an electroreductive synthesis,[^160^, ^161^] a one‐electron reduction,[^38^, ^39^, ^162^] and a polycondensation[^163^, ^164^, ^165^, ^166^] (Schemes 13, 14, 15, 16, 17, Figure 5, and Table 4). Stannylene is only used as an intermediate for the synthesis of oligostannanes due to its high reactivity.^[167]^ In contrast to polygermanes, metal‐catalyzed dehydropolymerization has been widely used because it can suppress the formation of cyclic oligomers as byproducts.[^150^, ^168^] Metal‐free dehydropolymerization is also available, however it requires high temperature to produce high molecular‐weight polystannanes.^[169]^

**TABLE 4:** Data and properties of polystannanes (SnR_2_)_*n*_.

	R	Synthetic method	*M*_n_	*M*_w_	Properties	References
**75**	^*n*^Bu	Metal‐catalyzed dehydropolymerization	20,300	66,900	Precursors to SnO_2_ and Sn metal Light absorption and emission	[143]
**76**	^*n*^Oct	Metal‐catalyzed dehydropolymerization	17,000	149,000	(*λ*_abs_ = 384 nm for **75**) (*λ*_abs_ = 402 nm for **76**) Photoreactivity Thermochromism	
**77**	^*n*^Bu	Wurtz‐type coupling	1,090,000	1,530,000	Light absorption (*λ*_abs_ = 380 nm) Photoreactivity	[141]
**78**	^*n*^Bu	Electroreductive synthesis	11,700	24,600	Light absorption (*λ*_abs_ = 379 nm for **78**)	[160]
**79**	^*n*^Oct	Electroreductive synthesis	5900	10,000	(*λ*_abs_ = 378 nm for **79**) Thermochromism	
**80**	^*t*^Bu and H	Metal‐catalyzed dehydropolymerization	16,700	56,000	Precursors to SnO_2_ and Sn metal Photoreactivity	[147]
**81**	^*n*^Hex and H	Metal‐catalyzed dehydropolymerization	20,000	48,200	Light absorption (*λ*_abs_ = 432 nm for **80**)	
**82**	H and Et	Metal‐catalyzed dehydropolymerization	− (insoluble)	−	(*λ*_abs_ = 436 nm for **81**) (*λ*_abs_ = 436 nm for **82** in film)	
**83**	O^*n*^Bu and H	Metal‐catalyzed dehydropolymerization	7000	12,000	(*λ*_abs_ = 448 nm for **83**) (*λ*_abs_ = 506 nm for **84**)	
**84**	O^*n*^Bu and Et	Metal‐catalyzed dehydropolymerization	4000	4400	(*λ*_abs_ = 450 nm for **85**)	
**85**	N(Me_3_Si)_2_ and H	Metal‐catalyzed dehydropolymerization	3800	4200		
**86**	Et	One‐electron reduction	3980	4820	Light absorption	[42, 162]
**87**	Me	One‐electron reduction	750	1120	(*λ*_abs_ = 368 nm for **86**)	
**88**	^*n*^Bu	One‐electron reduction	2030	2100	(*λ*_abs_ = 285 nm for **87**)	
**89**	^*n*^Hex	One‐electron reduction	2350	2770	(*λ*_abs_ = 289 nm for **88**) (*λ*_abs_ = 305 nm for **89**) Photoreactivity for the generation of polystannyl radicals	
**90**	Ph and ^*n*^Bu	Polycondensation	6600	18,800	Improved chemical stability to light and moisture for **90** and **91**	[165]
**91**	Ph and Me	Polycondensation	119,500	242,500		
**92**	Me and ^*n*^Bu	Polycondensation	28,700	82,100	Light absorption (*λ*_abs_ = 390 nm for **90**) (*λ*_abs_ = 395 nm for **91**) (*λ*_abs_ = 361 nm for **92**)	

It has been reported that the several unique properties of polystannanes. First, polystannanes have absorption bands in the visible region (350–420 nm) as well as conductivities originating from the more effective σ‐conjugation between catenated Sn atoms than polysilanes and polygermanes.^[170]^ The absorption behaviors can also be simulated by theoretical calculations.^[171]^ Second, some polystannanes showed a liquid crystalline phase at room temperature, and the light absorption behavior depended on the orientation.[^13^, ^172^, ^173^] Third, polystannanes are photoreactive, followed by the production of radicals by homolysis of weak Sn−Sn bonds upon irradiation of visible light,^[174]^ which can also be applied to photoinitiators, similarly to polygermanes. It is noted that the light stability of polystannanes can be improved by the addition of radical scavengers.^[175]^ Fourth, it is possible to form stable hypercoordination between polystannane main and side‐chains owing to the large atom size of tin.[^156^, ^157^, ^158^] Although the structures are similar to polysilanes and polygermanes, polystannanes have unique functions derived from the larger and heavier elements.

Dehydropolymerization of secondary hydrostannanes (R_2_SnH_2_) in the presence of zirconocene catalysts can be used for the preparation of polystannanes (Scheme 13). This method provided the moderate molecular‐weight poly (di‐*n*‐butylstannane) (**75**) (higher molecular‐weight fraction: *M*_n_ = 20,300, *M*_w_/*M*_n_ = 3.3) accompanied by ca. 18 wt% of lower molecular‐weight cyclic oligomers.^[143]^ From the TGA measurement, polymer **75** was thermally stable and no weight loss below 200°C. Interestingly, polymer **75** was photochemically depolymerized to a 2:1 mixture of cyclic pentamers and hexamers. Furthermore, poly (di‐*n*‐octylstannane) (**76**) was able to be applied to a thermochromic film between visible (*λ*_abs_ = 402 nm at 22°C) and ultraviolet (*λ*_abs_ = 378 nm at 47°C) regions. Moreover, thin films of polystannanes showed conductivities up to 0.3 S cm^−1^ after doped by exposure to SbF_5_ vapor. Recently, it was reported that the production of cyclic oligomers can be suppressed through the polymerization using Wilkinson's catalyst (RhCl(PPh_3_)_3_).^[150]^

Remarkably, the synthesis of extremely high molecular‐weight poly (di‐*n*‐butylstannane) (**77**) (*M*_n_ = 1,090,000, *M*_w_/*M*_n_ = 1.4) was achieved by a Wurtz‐type coupling reaction (Scheme 14).^[141]^ After the reaction for about 4 h, the maximum yield of polymer **77** including 20% cyclic byproducts was achieved. However, when the reaction time was extended to 5.5 h, polymer degradation occurred (90% cyclic byproducts). Based on this result, previous studies have failed to the polymeric stannanes probably due to the long reaction time.^[176]^ Furthermore, unlike dehydropolymerization, Wurtz‐type coupling requires a residual sodium quenching process, which might cause unexpected degradation of the polystannanes.

The electroreductive synthesis provided poly (di‐*n*‐butylstannane) (**78**) and poly (di*‐n*‐octylstannane) (**79**) in the one‐compartment cell equipped with a Pt cathode and an Ag anode (Scheme 15).^[160]^ Polymer **78** showed thermochromism; however, the changes were not discontinuous (*λ*_abs_ = ca. 376–379 nm) unlike the polygermane analog. It is known that the thermochromic behavior of σ‐conjugated polymers results from the conformational change of the main‐chain, which can be controlled by the interaction between the side‐chains. Therefore, non‐discontinuous thermochromism is probably due to the weak side‐chain interactions separated by long Sn–Sn bonds. In contrast, polymer **79** showed discontinuous thermochromic changes (*λ*_abs_ = ca. 377–385 nm) because of the strong side‐chain interactions between the longer alkyl chains.

The absorption behaviors of polystannanes can be modulated by the selection of substituents in the side‐chains. Five types of poly(diaryl)stannanes were prepared by dehydropolymerization of the corresponding secondary hydrostannanes with zirconocene catalysts (Figure 5).^[147]^ The poly(diaryl)stannanes, H(Ar_2_Sn)_*n*_H, (Ar = *p*‐^*t*^Bu‐C_6_H_4_ (**80**), *p*‐^*n*^Hex‐C_6_H_4_ (**81**), *o*‐Et‐C_6_H_4_ (**82**), *p*‐^*n*^BuO‐C_6_H_4_ (**83**), *o*‐Et‐*p*‐^*n*^BuO‐C_6_H_3_ (**84**), *p*‐(Me_3_Si)_2_N‐C_6_H_4_ (**85**)) exhibited absorption in the visible region (*λ*_abs_ = 430–506 nm) assigned to σ–σ* transitions. In particular, polymer **84** (*λ*_abs_ = 506 nm) had the longest absorption wavelength among group 14 σ‐conjugated polymers.

The one‐electron reduction of dichlorodialkylstannane proceeds with SmI_2_ in a THF–HMPA mixed solvent, and the process can be applied for polymerization (Scheme 16). The reaction was performed under mild conditions at room temperature for 24 h to afford poly(diethylstannane) (**86**) in 74% isolated yield.^[162]^ The reaction rate was accelerated by reflux, and the similar polymer was obtained after 5 h. Although a series of polystannanes with different side‐chains were prepared by the same reaction (R = Me (**87**), ^*n*^Bu (**88**), ^*n*^Hex (**89**)), their isolated yields were relatively low (6%–19%).[^42^, ^162^]

The polycondensation reaction between organotin compounds has been developed, and it is suitable for preparing alternating polystannanes (Scheme 17).[^165^, ^166^] By the stoichiometric reaction of tin dihydrides and tin diamides in diethyl ether or toluene, three types of alternating polystannanes were synthesized, [Ph_2_Sn‐*alt*‐Sn(*n*‐Bu)_2_]_*n*_ (**90**), [Ph_2_Sn‐*alt*‐SnMe_2_]_*n*_ (**91**), and [Me_2_Sn‐*alt*‐Sn(*n*‐Bu)_2_]_*n*_ (**92**).^[165]^ Interestingly, polymers **90** and **91** increased chemical stability to light and moisture in the solid state owing to the phenyl side‐chain, and there was no chemical change upon exposure ambient light for 5 days. In contrast, the alkylated polymer **92** degraded more rapidly accompanied by a noticeable color change of the powder from yellow to white mainly because of the formation of stannoxane.

#### 2.2.2 Tin‐containing polymers in the main‐chain

**SCHEME 18:**
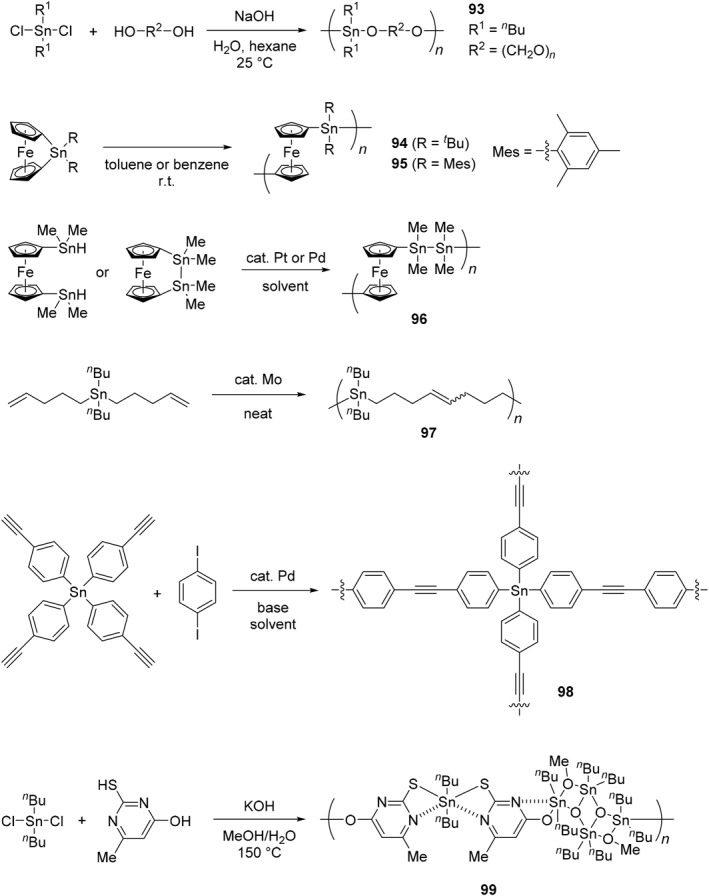
Synthetic methods of tin‐containing polymers in the main‐chain.

Similar to germanium‐containing polymers, tin‐containing polymers have been synthesized, and some different synthesis and properties have also been observed (Scheme 18 and Table 5). Organotin polyethers can be synthesized using an interfacial polymerization system involving the reaction of hydroxyl‐containing Lewis bases with organotin halides, and they are famous for showing their wide range of biological activities.^[183]^ For example, a high molecular‐weight organotin polyether (**93**) was prepared by the condensation reaction between di‐*n*‐butyltin dichloride and polyethylene grycol.^[177]^ It was revealed that the polymer has good solubility to water and ability to inhibit the growth of cancer cell lines from bone, prostate, colon, breast lung cancers.

**TABLE 5:** Data and properties of tin‐containing polymers in the main‐chain.

	Polymer type	Synthetic method	*M*_n_	*M*_w_	Properties	References
**93**	Polyether	Polycondensation	>100,000	−	Inhibition of cancer growth	[177]
**94**	Polymetallocene	Ring‐opening polymerization	560,000	900,000	High refractive index film	[80, 178]
**95**	Polymetallocene	Ring‐opening polymerization	1,050,000	1,350,000	Electrochemistry	
**96**	Polymetallocene	Metal‐catalyzed intermolecular dehydrogenative condensation Ring‐opening polymerization	54,180	95,350	Light absorption (*λ*_abs_ = 450 nm) Electrochemistry	[179]
**97**	Vinylene copolymer	Acyclic diene metathesis	36,000	76,000	−	[180]
**98**	Conjugated microporous polymer	Sonogashira–Hagihara coupling	−	−	High surface area (S_BET_ = 753 m^2^g^−1^) Emission	[181]
**99**	Coordination polymer	Solvothermal synthesis	−	−	−	[182]

The ROP is also applicable to tin‐bridged [1]ferrocenophanes, similarly to the germanium‐bridged analog.[^178^, ^184^] Poly(ferrocenylstannane)s having *t*‐butyl (**94**) and mesityl (**95**) side‐chains were synthesized by the ROP in toluene or benzene solution at room temperature.^[178]^ In the synthesis of polymer **94**, quantitative conversion of the monomer occurred within 6 h. In contrast, in the synthesis of polymer **95**, only 50% of the monomer was consumed even after 15 days. The ROP was also carried out in the solid state under heating conditions (150°C for the monomer of **94**, and 180°C for the monomer of **95**). These results indicate that the reactivity of the monomer of polymer **94** should be higher than that of the monomer of polymer **95**. In the absence of sterically bulky substituents in the monomer, the corresponding stanna[1]ferrocenophanes were not able to be isolated. This means that the presence of two sterically hindered substituents on tin is significant to avoid ring‐opening reactions during the monomer work‐up process. As described in germanium analogs, polymers **94** and **95** exhibited high refractive index *n* = 1.639 and 1.662 at 589 nm, respectively.^[80]^ The polymer having distannylene moieties (**96**) can be prepared by metal‐catalyzed intermolecular dehydrogenative condensation reaction or the ROP of the distannane‐bridged [2]ferrocenophane.^[179]^ The absorption band of polymer **96** was located around 450 nm which was longer than the DFT‐calculated absorption bands of dimeric (*λ*_abs_ = 419 nm) and trimeric (*λ*_abs_ = 423 nm) model fractions. This suggests that the conjugation can be extended through the polymer main‐chain composed of ferrocene and distannylene units.

Polycarbostannanes can be also synthesized by ADMET polymerization in the presence of molybdenum and tungsten catalysts.[^78^, ^180^] The moderate molecular‐weight polymer (**97**) was obtained by the molybdenum catalytic system.^[180]^ In the aryloxo tungsten system, it was revealed that the monomer worked as both the monomer and the cocatalyst species. Consequently, the polymer comparable to that obtained using the molybdenum catalytic system was produced.

Synthetic utility and unique reactivity of organotin compounds have been applied to the development of various polymeric materials.^[84]^ For instance, conjugated microporous polymers (CMPs) (**98**) were prepared as insoluble solids by a Sonogashira–Hagihara cross‐coupling reaction with tetrakis(4‐ethynylphenyl)stannane and diiodobenzene.^[181]^ Interestingly, the properties of the CMPs depended on the reaction condition, and they were isolated as the solids exhibiting emission from blue to yellow with large surface areas up to *S*_BET_ = 753 m^2^g^−1^ (*S*_BET_: Brunauer‐Emmett‐Teller surface area). Taking advantage of the reactivity of organotin compounds, Sn−C bonds were able to be cleaved by heating suspensions with chloroacetic acid in toluene. Through the process, the CMPs were converted to the linkers composed of di‐ or terphenyl fractions. Organotin compounds can form the higher coordination states and are readily used in the preparation of coordination polymers.[^185^, ^186^, ^187^] For example, a one‐dimensional infinite polymeric chain complex (**99**) was provided by the self‐assembly reaction of 4‐hydroxy‐2‐mercapto‐6‐methylpyrimidine, potassium hydroxide (KOH), and di‐*n*‐butyltin dichloride under solvothermal conditions (150°C).^[182]^ As applications of such tin‐containing coordination polymers, excellent activities towards different isolates have been revealed by antitumoral or biocide studies.[^188^, ^189^, ^190^, ^191^]

#### 2.2.3 Tin‐containing π‐conjugated polymers

**FIGURE 6:**
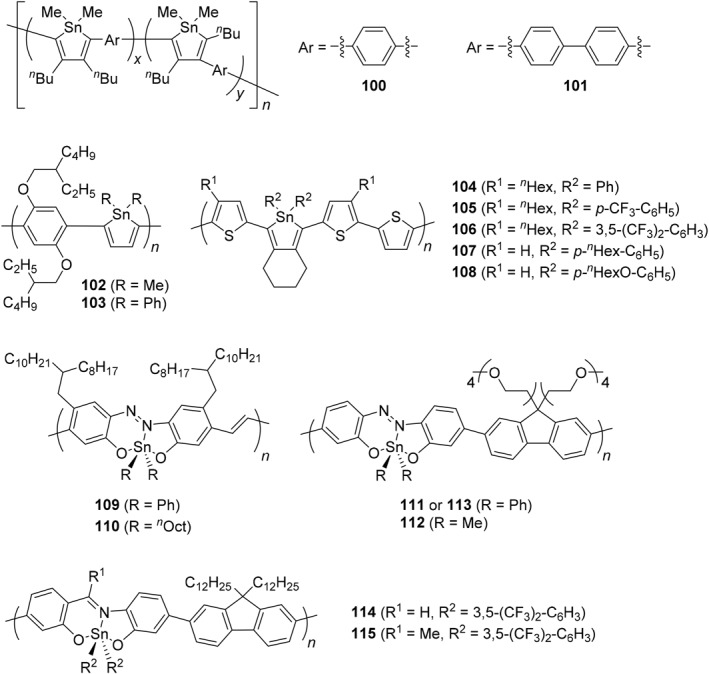
Structures of tin‐containing π‐conjugated polymers.

Synthetic methods have been developed to incorporate tin atoms into π‐conjugated systems, and the unique properties originating from tin atoms have also been revealed (Figure 6 and Table 6). Unlike germanium‐containing π‐conjugated polymers, the number of reports on tin‐containing π‐conjugated polymers is very limited due to the high reactivity of organotin π‐conjugated compounds towards metal‐catalyzed cross‐coupling reactions.^[200]^ Therefore, the synthetic ingenuity is required to prepare tin‐containing π‐conjugated polymers.

**TABLE 6:** Optical data and applications of tin‐containing π‐conjugated polymers.

	*M*_n_	*M*_w_	*λ*_abs_/nm	*λ*_PL_/nm	*Φ*_PL_/%	Application	References
**100**	2800	5900	410 (onset)	−	−	−	[192]
**101**	2700	7000	400 (onset)	−	−		
**102**	4100	9000	457	542	1.4	Chemosensor	[193]
**103**	4800	8600	491	−	−		
**104**	6800	17,000	536	−	−	−	[194, 195]
**105**	10,900	21,900	532	654	0.32		
**106**	10,300	21,500	522	655	<0.1		
**107**	4900	11,200	556	717	0.26		
**108**	5100	9600	560	716	<0.1		
**109**	9500	30,300	756	821	0.9	−	[196]
**110**	4600	11,000	752	811	1.5		
**111**	5300	7100	606	677	19	Vapochromism for **111**	[197]
**112**	6200	8200	614	682	24		
**113**	18,800	80,800	625	676	23.5	Vapochromism	[198]
						Thermochromism	
**114**	7100	8300	498	575, 618	49.1	Vapochromism for **115**	[199]
			505 (film)	591, 633 (film)	4.7 (film)	Thermochromism for **115**	
**115**	4700	6000	457	618	1.0		
			465 (film)	624 (film)	4.7 (film)		

The first synthesis of the tin‐containing π‐conjugated polymers was achieved by the post‐element transformation method.^[192]^ The stannole‐containing polymers (**100** and **101**) were obtained by the reaction of the corresponding organotitanium polymers and tin(IV) chloride followed by the treatment with methyllithium. The molecular weights of polymers **100** and **101** were *M*_n_ = 2800 and 2,700, respectively, and they were soluble in organic solvents. Polymers **100** and **101** exhibited broad absorption spectra and their onset values were 410 and 400 nm, respectively. Moreover, the LUMO energy levels of them were remarkably lower (−3.58 eV for **100** and −3.64 eV for **101**) than those of the well‐known π‐conjugated polymers (e.g., the thiophene‐containing polymer having similar structural units: −2.59 eV), and slightly higher than that of the germole‐containing polymer (−3.95 eV). Next, the alternating π‐conjugated polymers (**102** and **103**) were also prepared by the similar post‐element transformation method from the corresponding organotitanium polymers with diphenyl or dimethyltin chloride.^[193]^ In this case, the distinct absorption peaks were detected (*λ*_abs_ = 457 nm for **102** and *λ*_abs_ = 491 nm for **103**). Moreover, polymer **102** exhibited emission at 542 nm (*Φ*_PL_ = 1.4%). The reactivity of tin atoms is applicable to chemosensor. By the addition of tetrabutylammonium fluoride (TBAF) to the THF solution of polymer **102**, the solution color tuned from orange to dark red, and the emission was completely quenched. These results mean that the existence of fluoride ion can be detected by polymer **102** probably because of the bond formation between fluoride ion and tin. These findings suggest the potential of stannole‐containing polymers as chemosensors.

It is suggested that the reactivity of stannole can be modulated by the substituents on the tin atom, and it is expected that stannole‐containing π‐conjugated polymers can be synthesized even by metal‐catalyzed cross‐coupling reactions through the selection of the substituents. For instance, it was revealed that the 1,1‐diphenylstanole derivative was less reactive than the 1,1‐methyl and 1,1‐di‐*n*‐butylstannole derivatives in the same condition as the palladium‐catalyzed cross‐coupling reaction.^[194]^ From this insight, the diphenylstannole‐containing polymer (**104**) was able to be prepared by a Stille cross‐coupling polymerization. The absorption band of polymer **104** was observed at 532 nm, which was longer than that of the monomer (*λ*_abs_ = 441 nm). Based on this synthetic strategy, a series of diphenylstannole‐containing polymers (**105**–**108**) with electron‐donating and/or accepting substituents at phenyl moieties on the tin atoms were also obtained.^[195]^ The optical properties varied depending on the substituent effects and the planarity of the main‐chain. The emission was detectable; however, the intensity was low (*Φ*_PL_ < 0.4%) probably due to the heavy‐atom effect as well as long wavelength emission from the deep‐red to NIR regions (*λ*_PL_ = 654–717 nm).

Another approach to synthesize tin‐containing π‐conjugated polymers is to use hypervalent compounds. It is known that large and heavy tin atoms can stably form hypervalent states. Therefore, it is expected that the hypervalent tin moiety should be inactive to metal‐catalyzed cross‐coupling reactions. Indeed, PPV‐type π‐conjugated polymers (**109** and **110**) composed of hypervalent tin‐fused azobenzene (TAz) moieties were able to be prepared by the Stille‐cross coupling polymerization.^[196]^ Interestingly, polymers **109** and **110** showed both NIR absorption and emission properties (*λ*_abs_ = 756 nm, *λ*_PL_ = 821 nm, *Φ*_PL_ = 0.9% for **109**, and *λ*_abs_ = 752 nm, *λ*_PL_ = 811 nm, *Φ*_PL_ = 1.5% for **110**). As mentioned in the section on the hypervalent germanium polymers, the narrow energy gaps are caused by collaboration of azobenzene‐based π‐conjugated systems and hypervalent states of tin atoms. To the best of our knowledge, polymers **109** and **110** exhibit the longest wavelength absorption and emission among the PPV‐type π‐conjugated polymers. The π‐conjugated alternating copolymers (**111** and **112**) of TAz and fluorene units were also prepared by the same coupling polymerization.^[197]^ Surprisingly, the polymers **111** and **112** showed highly efficient fluorescence in the deep‐red region (*λ*_PL_ = 677 nm *Φ*_PL_ = 19% for **111**, and *λ*_PL_ = 682 nm, *Φ*_PL_ = 24% for **112**) despite the presence of heavy elements. Taking advantage of the reactive hypervalent tin center, a reversible vapochromic film can be fabricated from the solution of the polymers. The film of polymer **111** showed a reversible color change from blue (*λ*_abs_ = 612 nm) to purple (*λ*_abs_ = 597 nm) upon exposure to dimethyl sulfoxide (DMSO) vapor. The variation is derived from the reversible coordination number shift of tin center from five to six.^[135]^ On the other hand, the film of polymer **112** was inactive to the DMSO vapor. This is because that the phenyl‐substituted tin moiety of polymer **111** has higher binding ability of DMSO than the methyl‐substituted tin moiety of polymer **112**.

**FIGURE 7:**
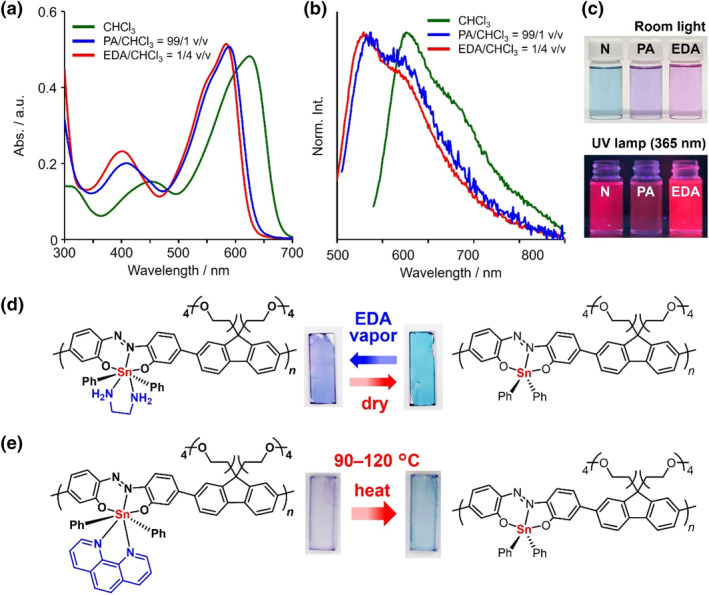
Optical properties of polymer **113**. (a) UV–vis absorption, (b) PL spectra, and (c) photographs in the mixed solvent of CHCl_3_ (N), propylamine (PA), and ethylene diamine (EDA) (1.0 × 10^−5^ M per repeating unit). (d) The reversible vapochromic film upon exposure of EDA vapor. (e) The irreversible thermochromic film. Reprinted with permission from ref ^[199]^. Copyright 2024 The Royal Society of Chemistry.

Hypervalent tin moiety can form not only six‐coordinated but also seven‐coordinated states. Owing to the chelate effect of a bidentate ligand, the seven coordination with ethylenediamine (EDA) had about 200 times higher binding constant than the six coordination with propylamine (PA) and DMSO.^[198]^ The higher molecular‐weight tin‐containing π‐conjugated polymer (**113**) was synthesized through the same method as polymer **111**. Polymer **113** showed a hypsochromic shift of the absorption and the PL bands by the coordinating solvent through the formation of the higher coordinated state of the tin center from five to six (PA) to seven (EDA) (Figure 7a–c). The film with polymer **113** exhibited clear and reversible vapochromism upon exposure to EDA vapor accompanied by the color change from greenish‐blue (*λ*_abs_ = 641 nm, *λ*_PL_ = 702 nm, five coordination) to purple (*λ*_abs_ = 598 nm, *λ*_PL_ = 697 nm, seven coordination) (Figure 7d). As the initial state, the film having seven coordinations can be prepared using the solid‐type bidentate ligand 1,10‐phenanthroline (phen). After preparing the film through a spin‐coating method with the solution of polymer **113** and phen, purple film (*λ*_abs_ = 587 nm, *λ*_PL_ = 651 nm) was obtained where the seven coordinations should be formed. Interestingly, the polymer film showed thermochromism (Figure 7e). The color change into greenish‐blue (*λ*_abs_ = 607 nm, *λ*_PL_ = 707 nm) occurred upon heating around 90–120°C, where the five coordinations should be induced by the release of phen. These results represent that heat‐history sensors can be realized.

The optical properties and binding ability of the tin center can be modulated by changing the π‐conjugated structure and substituents on the tin atom. The polymers (**114** and **115**) composed of hypervalent tin‐fused azomethine (TAm) moiety and electron‐withdrawing substituents on tin atoms were synthesized by the Stille cross‐coupling polymerization.^[199]^ Since the TAm moiety reduces the binding constant of the tin center to nucleophiles compared to the TAz one, the binding constant was enhanced by the introduction of trifluoromethyl groups as electron‐withdrawing substituents. In the solution, polymer **114** exhibited bright emission (*λ*_PL_ = 575, 618 nm, *Φ*_PL_ = 49.1%), whereas polymer **115** showed weak emission (*λ*_PL_ = 618 nm, *Φ*_PL_ = 1.0%). This is because that the methyl group at the azomethine moiety promotes the molecular motion at the π‐conjugated system. On the other hand, these polymers had similar emission efficiencies (*Φ*_PL_ = 4.7%) in the film by quenching and improving the emission for polymers **114** and **115**, respectively. Considering the higher chemical stability of the ketimine moiety against nucleophiles, polymer **115** was used as the stimuli‐responsive material. Consequently, film‐type thermochromic materials which can work below the freezing point (0°C) were obtained with polymer **115** by employing the seven‐coordinated tin and 2,2′‐bipyridyl. The thermochromism is irreversible, and it might be applicable to sensors which record unexpected temperature increases and provide quality assurance for materials such as vaccines or cells that must be stored at low temperatures. These optical changes and binding abilities are also predictable from theoretical calculations. These results mean that the π‐conjugated polymers composed of hypervalent tin moieties are a good candidate for designable stimuli‐responsive materials.

## 3 CONCLUSION AND OUTLOOK

Polymers containing germanium and tin are summarized. Even though germanium and tin compounds form similar structures, we have described that their reactivity and properties are greatly different. Polygermanes and polystannanes have promising properties for applications as thermochromic and conductive materials. In addition, their photoreactivity can be used for photoinitiators and lithographic techniques. In the carbon‐based polymers incorporating germanium and tin in the main‐chain, various structures can be constructed by the selection of monomers. The resulting polymers possess high refractive indices and antibacterial activities originating from heavy elements. Germanium‐containing π‐conjugated polymers have been applied not only as luminescent materials but also as charge carriers in organic solar cells. In the case of tin‐containing π‐conjugated polymers, not only optical properties but also reversible stimuli‐responsiveness can be achieved. Therefore, the introduction of heavy elements into polymer backbones is an effective strategy to bring out unique and diverse functions which are difficult to realize with conventional organic materials. Moreover, not only germanium and tin but also other heavy main‐group elements are expected to have their own unique characteristics. To effectively exploit the inherent properties of the elements, the exploration of novel molecular frameworks is also required. The continuous development of novel properties based on heavy elements will lead to the creation of smart materials.

## CONFLICT OF INTEREST STATEMENT

The authors declare no conflicts of interest.

## Data Availability

The data that support the findings of this study are available from the corresponding author upon reasonable request.
